# Efficacy and Safety of Tongxinluo Capsule as Adjunctive Treatment for Unstable Angina Pectoris: A Systematic Review and Meta-Analysis of Randomized Controlled Trials

**DOI:** 10.3389/fphar.2021.742978

**Published:** 2021-10-11

**Authors:** Pengqi Li, Qiqi Xin, Jiaqi Hui, Rong Yuan, Ya Wang, Yu Miao, Simon Ming-Yuen Lee, Sean X. Leng, Weihong Cong, BPNMI Consortium

**Affiliations:** ^1^ Laboratory of Cardiovascular Diseases, Xiyuan Hospital, China Academy of Chinese Medical Sciences, Beijing, China; ^2^ National Clinical Research Center for Chinese Medicine Cardiology, Xiyuan Hospital, Beijing, China; ^3^ State Key Laboratory of Quality Research in Chinese Medicine, Institute of Chinese Medical Sciences, University of Macau, Taipa, Macao, SAR China; ^4^ Division of Geriatric Medicine and Gerontology, Department of Medicine, Johns Hopkins University School of Medicine, Baltimore, MD, United States

**Keywords:** unstable angina pectoris, Tongxinluo capsule, Chinese medicine, efficacy, safety, systematic review, meta-analysis

## Abstract

Tongxinluo capsule (TXLC) is a commonly used Chinese medicine for unstable angina pectoris (UA). This article aimed to clarify the safety and efficacy of TXLC as an adjunctive treatment for UA. Two reviewers searched 7 databases from inception to August 2021, and performed literature screening and information extraction independently. The meta-analysis was implemented after evaluating the methodological quality of each randomized controlled trial (RCT) by the Cochrane Risk of Bias tool. Sensitivity analyses were conducted for testing the stability of the results, and the Begg and Egger tests were performed for any potential publication bias. After eligibility assessment, 42 RCTs with a total of 5,421 participants were included. Evidence showed that TXLC reduced the rate of cardiovascular events [RR = 0.29, 95% CI (0.19, 0.45), *p* < 0.00001, *I* ^*2*^ = 0%] {including cardiovascular mortality [RR = 0.16, 95% CI (0.03, 0.88), *p* = 0.03, *I* ^*2*^ = 20%], the incidence of acute myocardial infarction [RR = 0.27, 95% CI (0.13, 0.57), *p* = 0.0006, *I* ^*2*^ = 0%] and the occurrence of revascularization [RR = 0.28, 95% CI (0.15,0.54), *p* = 0.0001, *I* ^*2*^ = 0%]}, all-cause mortality [RR = 0.25, 95% CI (0.06, 0.99), *p =* 0.05, *I* ^2^ = 19%], recurrence of angina [RR = 0.25, 95% CI (0.11, 0.61), *p =* 0.002, *I* ^2^ = 0%], the number of ST-segment depression [MD = −0.45, 95% CI (−0.69, −0.20), *p =* 0.0005, *I* ^2^ = 0%], the summation of ST-segment depression [MD = −0.70, 95% CI (−1.08, −0.32), *p =* 0.0003, *I* ^2^ = 70%] and the hypersensitive C-reactive protein level [MD = −2.86, 95% CI (−3.73, −1.99), *p* < 0.00001, *I* ^2^ = 86%], increased the nitric oxide level [MD = 11.67, 95% CI (8.33, 15.02), *p* < 0.00001, *I* ^2^ = 33%], improved the electrocardiogram change [RR = 1.23, 95% CI (1.16, 1.30), *p* < 0.00001, *I* ^2^ = 0%] and the clinical efficacy in UA [RR = 1.26, 95% CI (1.21, 1.32), *p* < 0.00001, *I* ^2^ = 24%], and relieved the symptoms of angina pectoris {including chest pain or tightness [RR = 1.13, 95% CI (0.97, 1.32), *p* = 0.12, *I* ^2^ = 30%], palpitations [RR = 1.47, 95% CI (1.18, 1.84), *p* = 0.0007, *I* ^2^ = 0%], shortness of breath [RR = 1.53, 95% CI (1.24, 1.88), *p* < 0.0001, *I* ^2^ = 0%], and asthenia [RR = 1.69, 95% CI (0.83, 3.43), *p* = 0.15, *I* ^2^ = 90%]}. The most common adverse effect was gastrointestinal symptoms which could be relieved and eliminated through dose reduction, medication time adjustment and symptomatic remedy. Collectively, TXLC was effective and considerably safe for UA. However, due to the unavoidable risk of bias, these results must be interpreted with caution and further verified by large-scale and high-quality RCTs.

**Systematic Review Registration:** www.crd.york.ac.uk/PROSPERO/, identifier CRD42021232771.

## Introduction

The World Health Organization (WHO) reported that the global number of individuals with cardiovascular diseases (CVDs) had doubled from 271 million in 1990 to 523 million in 2019. In China, the number of CVD patients reached approximately 330 million in 2019. Ischemic heart disease (IHD), the most common CVD, was currently the largest international cause of death, bringing heavy economic burdens and health threats to the world (Roth et al., 2020; The Writing Committee of the Report on Cardiovascular Health Diseases in China., 2020). As one of the most common and typical IHD, unstable angina pectoris (UA) was manifested as a significant exacerbation of angina symptoms (Cannon et al., 2001), and often progressed rapidly, even to acute myocardial infarction (AMI) or sudden death.

Local coronary artery lesions including unstable plaques, thrombosis, vasospasm, and intravascular inflammation are regarded as the pathological basis of UA, which cause vascular stenosis or blockage and lead to myocardial ischemia. Accordingly, the conventional treatments (CTs) for UA mainly include anti-platelet, anti-coagulation, blood lipids regulation, angina control, and anti-myocardial ischemia. However, the currently available treatment regimens for UA represent an unmet medical need, such as the clinical resistance to antiplatelet agents or lipid-lowing drugs (Helgeson et al., 1994; Chessman et al., 2004; Serebruany et al., 2005; Münzel et al., 2011) and the adverse effects during long-term medication. Given the great variability in individual efficacy and poor patient compliance of the currently available treatment regimens, it is difficult to obtain satisfactory therapeutic effects against UA. Therefore, finding potential approaches for alleviating limitations on CTs of UA is warranted. Tongxinluo capsule (TXLC), a Chinese medicinal product composed of *Panax ginseng* C.A.Mey. (Ren Shen), *Hirudo nipponica* Whitman (Shui Zhi), *Scolopendra subspinipes mutilans* L. Koch (Wu Gong), *Eupolyphaga sinensis* Walker (Tu Bie Chong), *Buthus martensii* Karsch (Quan Xie), *Cryptotympana pustulata* Fabricius (Chan Tui), *Paeonia lactiflora* Pall. (Chi Shao), *Dryobalanops aromatica* C.F.Gaertn. (Bing Pian), *Santalum album* L. (Tan Xiang), *Boswellia carterii* Birdw. (Ru Xiang), *Dalbergia odorifera* T.C.Chen (Jiang Xiang), *Ziziphus jujuba* Mill. (Suan Zao Ren), etc., is widely used in China and has been recommended by several guidelines and expert consensuses for the treatment of angina pectoris [e.g., the Guidelines for Rational Use of Drugs for Coronary Heart Disease (Second Edition): TXLC can reduce the adhesion of platelets to collagen fibers and significantly relieve clopidogrel resistance during DAPT treatment]. In high-performance liquid chromatography analysis, the similarity of the fingerprints of each batch of TXLC was above 95%, indicating that the product quality was stable and controllable (Meng et al., 2014; Li Q. et al., 2018). Clinical and laboratory researches have been conducted since 1995, indicating that TXLC plays a positive role in enhancing cardiac systolic function, protecting the vascular endothelium, delaying the progression of atherosclerosis, preventing coronary embolism after PCI in patients with AMI, reducing vascular endothelial damage, preventing heart failure caused by pressure overload and regulating cytokine levels with multiple targets (Ma et al., 2009; Chen et al., 2016; Wang et al., 2019; Zhang et al., 2019; Li et al., 2020). The previous meta-analyses showed that TXLC had a good secondary preventive effect against AMI, the addition of TXLC to conventional western medicine might prevent the recurrence of restenosis and cardiovascular events in patients with coronary heart disease after PCI. It also effectively reduced the symptoms of angina pectoris in Cardiac Syndrome X and seemed to be more effective than β-blockers in the treatment of angina pectoris (Jia and Leung., 2015; Mao et al., 2015; Li Q. et al., 2018; Mao et al., 2018). At present, TXLC is widely used for UA as an adjuvant treatment. Nevertheless, some adverse effects, such as digestive tract reactions, bleeding gums and blood biochemical changes, have been reported (Xu, 2013; Xu and Shao., 2020). Given that, the efficacy and safety of TXLC for UA need to be reassessed, for providing new inspiration for UA’s current therapeutic regimen.

## Methods

### Protocol and Registration

The systematic review protocol was registered in the International Prospective Register of Systematic Reviews (PROSPERO) (No. CRD42021232771). All projects, including the design, implementation, analysis, and report, were determined following the PRISMA guidelines (Page et al., 2021). See Supplementary Material S1 for the PRISMA 2020 Checklist.

### Search Strategies

Two reviewers (PL and JH) independently completed the literature search without restrictions on language, race or literature scope. The search aimed at all related studies published on Cochrane Central Register of Controlled Trials (CENTRAL), PubMed, EMBASE Database, China National Knowledge Infrastructure (CNKI), Chinese Biomedical Literature Service System (SinoMed), Wanfang Database and Chinese Scientific Journal Database (VIP) as of August 31, 2021. “Angina, Unstable” was used as the Medical Subject Heading and matched with corresponding free words for enhancing accuracy, and various expressions of “tong xin luo” were connected with truncation characters for describing the intervention part. Given the discrepancy between databases, the keywords were adjusted flexibly for “randomized controlled trial, RCT or semi-randomized controlled trial”. Finally, all retrieval expressions were formed by logically connecting AND or OR. For example, EMBASE Database was searched as (‘tongxinluo capsule’: ab, ti OR ‘tong xin luo*’: ab, ti OR ‘tong-xin-luo*’: ab, ti OR ‘txl*’: ab, ti OR ‘tongxinluo*’: ab, ti) AND (‘random’: ab, ti OR ‘placebo’: ab, ti OR ‘double-blind’: ab, ti). See Supplementary Material S2 for the complete search strategies.

### Study Selection Criteria

#### Study Design and Participants

All randomized controlled trials (RCTs) or semi-randomized controlled trials evaluating the efficacy or safety of TXLC for the treatment of UA were included regardless of blinding. The sample sizes of selected studies were all greater than 100. There were no restrictions on participants’ gender, race, age, nationality, course, or severity of disease. Participants had to meet available diagnostic criteria such as the “2000 WHO diagnostic criteria for unstable angina pectoris”, “2000 Chinese Medical Association recommendations for diagnosis and treatment of UA”, “1979 WHO nomenclature and diagnostic criteria of IHD” and other standards or consensuses, and accompanied by recent angina pectoris attacks and electrocardiogram (ECG) ischemic ST-T changes. Patients in any of the following conditions were excluded: severe disease of the brain, lung, liver, kidney, or other organs; active bleeding, infections, tumors, or immune system diseases; history of drug allergic reactions; pregnancy or lactation; and chest pain from other etiologies at the time of the study.

#### Interventions

Patients treated with CTs, including isosorbide dinitrate, low molecular weight heparin, β-blockers, aspirin, angiotensin-converting enzyme inhibitors and angiotensin receptor blockers, were classified in the control group, while the intervention of the trial group was TXLC combined with CTs. Patients with hyperlipidemia, diabetes, hypertension or certain complications were treated accordingly. Except for TXLC, trials involving any other traditional Chinese medicine interventions (such as qigong, acupuncture, other herbs, and moxibustion) were excluded.

#### Outcome Measures

Preset primary or secondary outcome indicators must have been reported in the included trials. The primary outcome indicators were defined as the incidence of all-cause mortality, the incidence of cardiovascular events, and adverse effects. The incidence of cardiovascular events was a comprehensive outcome of AMI, cardiac death and revascularization [including percutaneous coronary intervention (PCI), percutaneous transluminal coronary angioplasty (PTCA) and coronary artery bypass grafting (CABG)]. Any adverse effect and withdrawal of patients due to intolerances was recorded.

Secondary outcome indicators comprised the relapse of angina, the number of ST-segment depression (NST), summation of ST-segment depression (∑ST), ECG improvement, clinical efficacy in UA, symptom improvement (chest pain or tightness, palpitation, shortness of breath and asthenia), hypersensitive C-reactive protein (hs-CRP) level, and level of nitric oxide (NO). The ECG improvement was defined as a recovery of ST-segment depression exceeding 0.05 mV. The clinical efficacy of UA was considered meeting one of the following conditions as effective (otherwise it was invalid): 1) the frequency, duration or nitroglycerin dose of UA decreased by more than 50% compared with previously; 2) Canadian Cardiovascular Society classification of angina pectoris improved 1 level or above; and 3) cardiac load grew without increasing angina frequency. Outcomes were evaluated at the point of longest follow-up time when more than one follow-up time was mentioned.

### Data Extraction and Quality Assessment

All records were imported into reference management software (EndNote X7) to eliminate duplicates. Study eligibility was independently assessed by 2 reviewers (PL and QX) according to the inclusion/exclusion criteria. Irrelevant literatures, such as reviews and pharmacological trials, were eliminated by reading titles and abstracts. Full texts were read before confirming inclusion. The reviewers further clarified studies with unclear titles or abstracts for potential inclusion (PL and QX). If repeated data were published by the same author across studies, the latest published or the one with the largest sample size was selected. To facilitate data statistics, a standard form was used for data extraction which including the following: 1) study ID, 2) sample size, 3) baseline characteristics of participants (sex, age, etc.), 4) interventions (dosage of administration), 5) duration of therapy, 6) UA diagnostic criteria, and 7) outcomes and adverse effects. Authors of the original studies were consulted for unclear or missing information when necessary. Any disagreement was resolved through discussion between two reviewers or with another author.

### Risk of Bias Assessment

Two investigators (PL and QX) independently assessed the quality of the included studies according to the Cochrane Collaboration tool, which included 7 areas of low, high and undefined risks: 1) random sequence generation, 2) allocation concealment, 3) blinding of participants and personnel, 4) blinding of outcome assessment, 5) incomplete outcome data, 6) selective reporting, and 7) other bias. An item was judged as “unclear” when it encountered ambiguous information or could not be determined to be “high” or “low”.

### Statistical Synthesis and Analysis

Meta-analysis was performed with the Review Manager Software package (RevMan, v.5.3; The Cochrane Collaboration). The relative risk (RR) of dichotomic variables and the mean difference (MD) of the continuous variables were calculated with 95% CIs, and the results were presented as forest maps. Skewed data and nonquantitative data were presented with descriptions. All reported *p* values were two-tailed and considered statistically significant when *p* < 0.05. The *I* ^*2*^ statistic was applied for heterogeneity assessment. *I* ^*2*^ ≥ 50% showed significant heterogeneity, with a random effect model being applied; otherwise, a fixed effect model was adopted instead. Sensitivity analysis was used to test the robustness of the results. If an indicator was reported in more than 10 included trials, potential publication bias would be assessed by an inverted funnel plot (Sterne and Egger., 2001). Meanwhile, the Begg rank correlation (Begg and Mazumdar., 1994) and Egger regression asymmetry test (Egger et al., 1997) performed by STATA v.12.0 (Stata Corp LP, College Station, TX, United States) were used to assess the dissymmetry degree of the funnel plot (*p* < 0.05).

## Results

### Search Results

**FIGURE 1:**
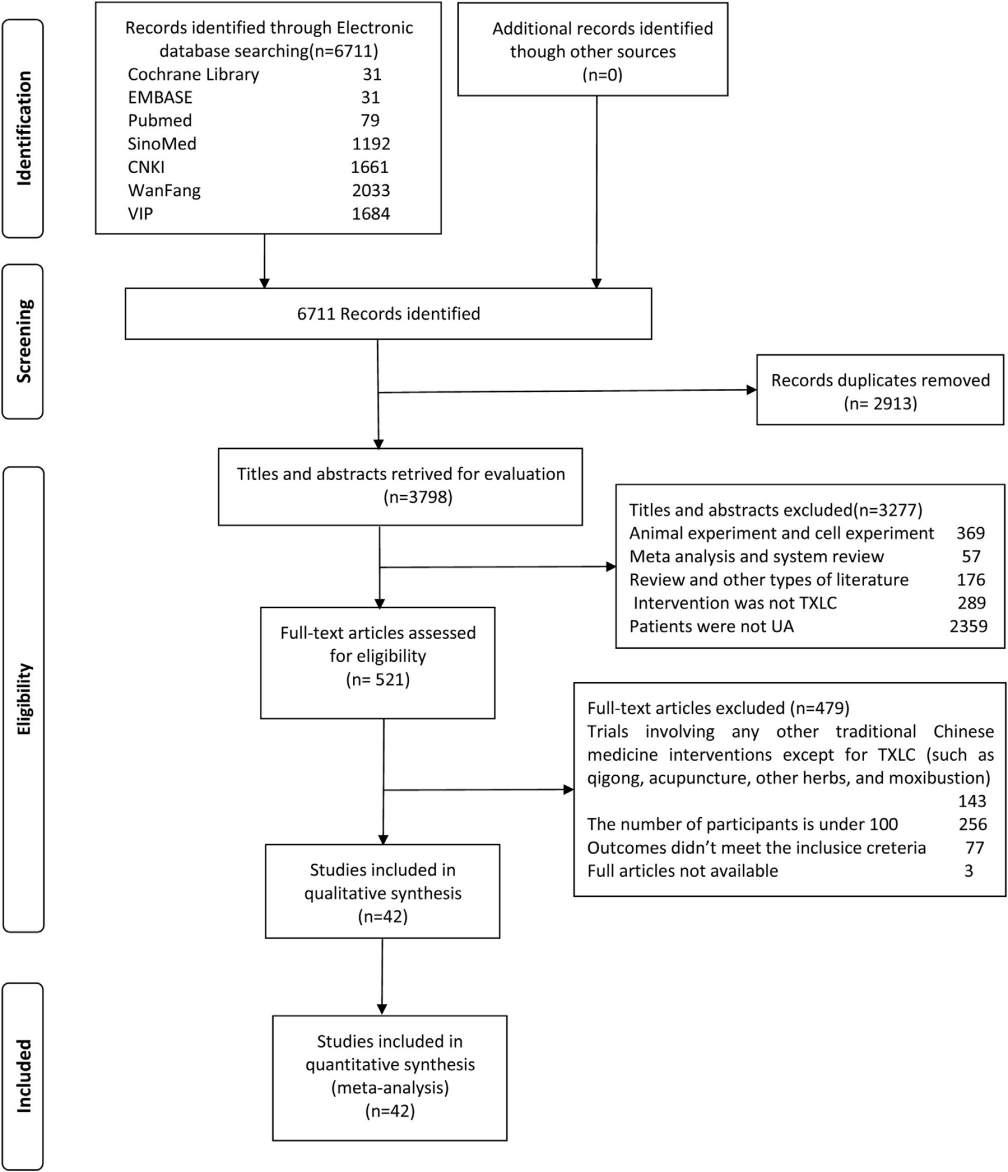
Flow chart of literature search.

A total of 6,711 studies were identified from preliminary searches according to the above retrieval strategy. After removing 2,913 duplicates and 3,277 substandard studies by browsing titles and abstracts, 521 papers were retained for further assessment. After screening based on the inclusion and exclusion criteria, 42 standard-compliant RCTs were included in the final analysis. Figure 1 presents the detailed screening flow of eligible studies.

### Characteristics of the Included Studies

Ultimately, 5,421 patients from 42 trials (Cai and Li., 2010; Chang et al., 2018; Chang and Zhao, 2004; Chen and Li, 2009; Cui, 2008; Ding et al., 2013; Du, 2016; Gao et al., 2002; Hao, 2015; Hui et al., 2018; Jiang et al., 2019; Li Q. et al., 2018; Li, 2013; Liu and An., 2016; Liu, 2011; Luo, 2013; Ma et al., 2011; Ren et al., 2018; Shi, 2013; Song et al., 2008; Sun et al., 2011; Tian and Xu., 2005; Tian et al., 2007; Wang and Li., 2007; Wang, 2017; Wang et al., 2013; Wang et al., 2010; Wang, 2015; Wang et al., 2009; Wang et al., 2012; Wu, 2011; Wu S. J. et al., 2006; Wu et al., 2010; Xin et al., 2008; Xing, 2013; Yang, 2008; Yang et al., 2019; Yu and Chen., 2015; Yu and Hu., 2012; Yuan, 2019; Zhang and Li, 2009; Zhou, 2013) (published between 2002 and 2019) were included. All selected studies were single center, parallel design, and conducted in China. There were 2,867 participants in the trial group (TXLC combined with CTs) and 2,554 participants in the control group (CTs only). The age of participants ranged from 30 to 90 years old. Six trials (Yang, 2008; Liu., 2011; Sun et al., 2011; Shi., 2013; Hao., 2015; Chang et al., 2018) had more female participants than males, 4 trials (Chang and Zhao, 2004; Wu S. J. et al., 2006; Song et al., 2008; Ren et al., 2018) did not report the gender ratio, and the remaining trials had more male participants. Both trial and control groups received CTs including nitrate, aspirin, statins, low molecular weight heparin, β-blockers, and so on. TXLCs were purchased from Shijiazhuang Yiling Pharmaceutical Co., Ltd., with a specification of 30 capsules per box, 0.26 g per pill. For the dosages of TXLC taken in the included trials, 4 capsules three times per day was the most frequently dosage used, which was implemented in 21 studies (Chen and Li, 2009; Cai and Li, 2010; Ding et al., 2013; Hao, 2015; Du, 2016; Liu and An., 2016; Ren et al., 2018; Jiang et al., 2019; Shi, 2013; Sun et al., 2011; Tian et al., 2007; Wang and Li, 2007; Wang, 2017; Wang et al., 2013; Wang, 2015; Wang et al., 2009; Wang et al., 2012; Wu et al., 2010; Xin et al., 2008; Xing, 2013; Zhang and Li, 2009). In addition, there were 9 cases (Chang and Zhao, 2004; Cui, 2008; Yang., 2008; Cai and Li., 2010; Wang et al., 2010; Zhou., 2013; Du., 2016; Chang et al., 2018; Hui et al., 2018) of 3 capsules three times per day, 4 cases (Yu and Hu., 2012; Li., 2013; Yu and Chen., 2015; Li Q. et al., 2018) of 2 capsules three times per day, 3 cases (Liu., 2011; Jiang et al., 2019; Yang et al., 2019) of 4 capsules twice per day, and 1 case (Song et al., 2008) of 2 capsules twice per day, and dosages in the rest of the studies were adjusted between the maximum and the minimum according to the actual conditions. The trial duration ranged from 2 weeks to 1 year. Seventeen studies (Cai and Li., 2010; Chang et al., 2018; Du., 2016; Gao et al., 2002; Hui et al., 2018; Li Q. et al., 2018; Li., 2013; Liu and An., 2016; Shi., 2013; Wang., 2017; Wang et al., 2013; Wang., 2015; Wang et al., 2012; Xing., 2013; Yang., 2008; Yu and Hu., 2012; Zhang and Li., 2009) mentioned the course of disease, ranging from 2 days to 21 years. Eight studies (Song et al., 2008; Xin et al., 2008; Wang et al., 2009; Cai and Li., 2010; Wu., 2011; Li., 2013; Wang et al., 2013; Hui et al., 2018) reported comorbidities in UA patients, including at least one case of diabetes, hypertension, or hyperlipidemia. The details of all studies were summarized in Table 1. See the TXLC quality control data of all included studies in Supplementary Material S3.

**TABLE 1:** The basic information of the 42 included articles (sorted by the first letter of author’s name).

Studies	Sample size (T/C)	Sex M/F	Age, Mean ± SD (year)	Intervention			Course of treatment	Diagnostic criteria	Outcomes
				T	TXLC dosage	C			
Cai and Li (2010)	120 (61/59)	73/47	NR	TXLC + CT	4 capsules, Tid	CT (isosorbide dinitrate, diltiazem hydrochloride tables, metoprolartrate tables, sublingual nitroglycerin if angina attach, no details)	4 weeks	2000 CMA recommendations for diagnosis and treatment of UA	(6) (8) (9)
Chang et al. (2018)	108 (54/54)	53/55	T	TXLC + CT	3 capsules, Tid	CT (aspirin 100 mg Qd, atorvastatin 20 mg Qd, isosorbide mononitrate 40 mg Qd, subcutaneous injection of enoxaparine 0.6 mg Q12h if necessary)	30 days	2007 CMA Guidelines for the diagnosis and management of UA and non-ST-segment elevation myocardial infarction	(11) (12)
			57.2 ± 2.8						(13) (14)
			C						(15) (17)
			57.7 ± 2.4						
Chang and Zhao (2004)	114 (68/46)	NR	T 65 ± 4 C 64 ± 6	TXLC + CT	3 capsules, Tid	CT (isosorbide dinitrate, aspirin, calcium antagonists, β-blocker, no details)	6 weeks	WHO Diagnostic criteria for UA	(10)
Chen and Li (2009)	118 (60/58)	79/39	NR	TXLC + CT	4 capsules, Tid	CT (aspirin 100 mg, Qd; simvastatin 10 mg, Qd; subcutaneous injection of LMWH 5000U, Q12h; metoprolol)	4 weeks	2000 CMA recommendations for diagnosis and treatment of UA (except for variantangina vectoris)	(6) (10)
Cui (2008)	144 (76/68)	82/62	T:55 ± 2	TXLC + CT	3 capsules, Tid	CT (β-blocker, calcium antagonists, aspirin, etc.; nitroglycerin, ivgtt; LMWH Calcium Injection, iH, 5–7 d)	1 month	1979 WHO nomenclature and diagnostic criteria of IHD	(6) (10)
			C:55 ± 3						
Ding et al. (2013)	120 (60/60)	77/43	NR	TXLC + CT	4 capsules, Tid	CT (aspirin, 100 mg, Qd; metoprolol 12.5 mg, Bid; nitroglycerin for angina attack 0.5–1 sublingual; simvastatin 10 mg, Qd; LMWH 5000U, iH, Q12h)	4 weeks	2000 WHO Diagnostic criteria for UA	(10) (11)
Du (2016)	100 (50/50)	51/49	T	TXLC + CT	4 capsules, Tid	CT (ACEI; nitrates; lipid-altering drugs; β-blocker; aspirin, 100 mg, Qd)	4 weeks	2000 WHO Diagnostic criteria for UA	(6) (10) (11)
			63.3 ± 5.4						
			C						
			62.9 ± 5.1						
Gao et al. (2002)	100 (60/40)	63/37	NR	TXLC + CT	2–4 capsules, Tid	CT (nitroglycerin, metoprolol, aspirin, etc., no details)	4 weeks	WHO Nomenclature and diagnostic criteria of IHD and the clinical research guidelines for new traditional Chinese medicines for the treatment of chest pain formulated by the Ministry of Health in 1993	(10)
Hao (2015)	110 (55/55)	53/57	T	TXLC + CT	4 capsules, Tid	CT (aspirin, nitrates, metoprolol, simvastatin, etc., no details)	2 months	Diagnostic criteria of UA in “Internal medicine”	(10)
			48.1 ± 3.8						
			C						
			49.3 ± 3.3						
Hui et al. (2018)	100 (50/50)	55/45	T	TXLC + CT	3 capsules, Tid	CT (aspirin, β-blocker, statins, nitrates, atients with diabetes were also given hypoglycemic therapy, isosorbide mononitrate for angina attack)	2 weeks	Diagnostic criteria for UA	(11)
			56.20 ± 6.75						
			C						
			56.56 ± 6.32						
Jiang et al. (2019)	160 (80/80)	90/70	T	TXLC + CT	4 capsules, Tid	CT (antiplatelet aggregation, calcium antagonist and anticoagulant therapy. astatin tablets 20 mg/times, Qd, daily bedtime oral)	3 months	Relevant standards formulated by the South China International Cardiovascular Symposium	(16)
			58.5 ± 6.4						
			C						
			59.1 ± 6.2						
Li Q. et al. (2018)	128 (64/64)	74/54	T:68.11 ± 7.29	TXLC + CT	2 capsules, Tid	CT (conventional treatment and a torvastatin calcium tablets 20 mg, Qd)	2 months	2009 edition of “Coronary Heart Disease with Integrated Traditional Chinese and Western Medicine”	(16)
			C:68.11 ± 7.29						
Li (2013)	110 (55/55)	81/29	T:55.4 ± 9.6	TXLC + CT	2 capsules,Tid	CT (atorvastatin, 20 mg, Qd; oral nitrates, β-receptor blockers, calcium antagonists, anti-platelet aggregation drugs and LMWH, etc.)	8 weeks	Diagnostic criteria for UA in the 1979 WHO standards and the standards of the National Symposium on the Diagnosis and Treatment of UA in August 2000	(11)
			C:57.0 ± 9.2						
Liu and An (2016)	160 (80/80)	101/59	51.1 ± 1.4	TXLC + CT	4 capsules, Tid	CT (sublingual nitroglycerin; aspirin antiplatelet therapy; heparin anticoagulation therapy; thrombolysis; β-blockers (propranolol) and ACEI (angiotensin II), attovastatin calcium tablets, 20 mg, Qd)	3 months	The relevant diagnostic criteria for coronary heart disease and angina pectoris formulated by the WHO; all are diagnosed as UA of coronary heart disease through clinical symptoms, laboratory examinations, and imaging data	(6)
Liu (2011)	102 (51/51)	47/54	NR	TXLC + CT	4 capsules, Bid	CT (conventional treatment and LMWH 5000U, iH, Q12h, continuous use of 5–7 d)	4 weeks	The diagnostic criteria for unstable myocardial infarction in the “Guidelines for the Diagnosis and Treatment of Elevated Myocardial Infarction” formulated by the Cardiovascular Branch of the CMA in 2007	(8) (9)
Luo (2013)	120 (60/60)	63/57	T:58.88 ± 14.37	TXLC + CT	2–4 capsules, Tid	CT [rest, oxygen inhalation, low-fat diet, give nitrate vinegar drugs, aspirin, lipid-lowering drugs, calcium antagonists, LMWH, metoprolol tartrate (start from the minimum dose of 6 t 25 mg, Bid, every 1–2 weeks to gradually increase, and finally increase to the target value of 50–150 mg, Qd, for 14 consecutive days)]	3 months	“Naming and Diagnostic Standards for Coronary Heart Disease” developed by WHO	(11)
			C:59.12 ± 15.01						
Ma et al. (2011)	318 (159/159)	194/124	60.6 ± 12.8	TXLC + CT	3–4 capsules, Tid	CT (aspirin, nitroglycerin, heparin, calcium antagonists, β-blockers, no details)	NR	Diagnosis based on the characteristics of angina pectoris and the dynamic evolution of the S-T segment of the ECG at the onset (s-T segment downward shift ≥0.1 mv)	(1) (2) (3) (4) (5) (6)
Ren et al. (2018)	100 (50/50)	NR	NR	TXLC + CT	4 capsules, Tid	CT (nitrate drugs, β-blockers, aspirin orally, 100 mg, Qd)	4 weeks	Evidence for the diagnosis of UA	(6) (11)
Shi (2013)	112 (56/56)	45/67	65.38 ± 10.57	TXLC + CT	4 capsules, Tid	CT (rest on bed, low-salt diet, low-flow oxygen inhalation, etc. nifedipine tablets, 10 mg, Tid; aspirin enteric-coated tablets 112 mg, Qd; angiotensin converting enzyme inhibitor benazepril 5 mg, Qd; trimetazidine, 20 mg, Tid; isosorbide dinitrate tablets 10 mg, Tid; take isosorbide dinitrate tablets when angina pectoris attacks, 10 mg/time)	14 days	Diagnostic criteria for UA developed by experts from the ACC and the American Association of Cardiology (AHA)	(6) (10) (11)
Song et al. (2008)	176 (106/70)	NR	NR	TXLC + CT	2 capsules, Bid	CT (nitroglycerin 5mg, added to 5% glucose injection 250 ml intravenous infusion, first start at 10 μg/min, increase by 5–10 μg every 15 min, maintain the systolic blood pressure at about 100 mmHg)	2 weeks	Standards established by WHO in 1979	(6) (10) (11)
Sun et al. (2011)	128 (66/62)	62/66	68.26 ± 10.17	TXLC + CT	4 capsules, Tid	CT (aspirin 100 mg, Qd; atorvastatin 20 mg, Qn; isosorbide mononitrate 20 mg, Bid; oral ACEI and calcium antagonists, β-blockers. intravenous nitrates and subcutaneous injection of LMWH if necessary)	4 weeks	WHO recommended diagnostic criteria for UA	(16)
Tian and Xu (2005)	118 (77/41)	82/36	NR	TXLC + CT	TXLC group 1: 45 cases, 2 capsules, Tid; TXLC group 2: 32 cases, 4 capsules, Tid	CT (isosorbide 10 mg, tid; enteric-coated aspirin 0.1 g, Qd; captopril 6.25–25 mg, Tid; and add β-blockers or calcium antagonists, statins lipid-lowering drugs, intravenous nitrates and subcutaneous injection of LMWH if necessary)	8 weeks	1997 WHO Diagnostic criteria for IHD	(5) (6)
Tian et al. (2007)	120 (60/60)	69/51	57.4 ± 4.7	TXLC + CT	4 capsules, Tid	CT (Conventional coronary artery dilation, anticoagulation, and oxygen consumption reduction therapy)	4 weeks	WHO standard for UA	(6)
Wang and Li (2007)	180 (90/90)	124/56	T:56 ± 6	TXLC + CT	4 capsules, Tid	CT [enteric-coated aspirin (changed to 100 mg/d after 300 mg/d, 3 days), nitrate, β-blockers, LMWH sodium (5000 IU subcutaneous injection, Q12h) (LMWH for 1 week)]	8 weeks	Selection criteria: 1. initial exertional angina pectoris; 2. deteriorating exertional angina pectoris; 3. resting angina pectoris; 4. angina after infarction. At the same time: 1.96 h of sudden exacerbation of angina, activity tolerance decreased significantly; 2. spontaneous angina attack at least once within 24 h; 3. ST-segment moved down more than 1 mm at the time of the attack, and it recovered significantly after the attack was relieved	(6) (11)
			C:56 ± 7						
Wang (2017)	120 (60/60)	62/58	T:63.1 ± 5.2 C:62.8 ± 4.3	TXLC + CT	4 capsules, Tid	CT (nitrate drugs, ACEI, β-blockers, aspirin, 100 mg/time)	4 weeks	All patients meet the clinical diagnostic criteria for UA established by the Cardiovascular Branch of the CMA in 2000;	(6) (11)
								ASA Cardiac Function Classification I∼II	
Wang et al. (2013)	150 (100/50)	85/65	T:74.28 ± 5.14	TXLC + CT	4 capsules, Tid	CT (antiplatelet aggregation and anticoagulant drugs, antiangina drugs; in special circumstances, quick-acting anti-angina pectoris can be added temporarily)	4 weeks	The diagnostic criteria for UA in the 2007 “Guidelines for the Diagnosis and Treatment of UA and Non-ST Segment Elevation Myocardial Infarction”. The angina pectoris classification adopts the angina pectoris classification of the CCS	(4) (10) (11) (12) (13) (14) (15)
			C:72.80 ± 4.98						
Wang et al., 2010	110 (56/54)	74/36	63.3 ± 7.2	TXLC + CT	3 capsules, Tid	CT (aspirin + simvastatin + nitrate)	12 weeks	UA risk stratification of Brauwald in 1989	(11)
Wang (2015)	100 (50/50)	62/38	T:75.3 ± 2.7	TXLC + CT	4 capsules, Tid	CT (antiangina drugs, antiplatelet aggregation drugs and anticoagulant drugs)	1 month	clinical diagnostic criteria for UA	(11)
			C:74.8 ± 3.1						
Wang et al. (2009)	126 (66/60)	88/38	T:54.3	TXLC + CT	4 capsules, Tid	CT (isosorbide, 10 mg, Tid)	2 months	The naming and diagnostic criteria of IHD developed by WHO	(6) (11)
			C:53.8						
Wang et al. (2012)	144 (72/72)	80/64	T:68.6 ± 8	TXLC + CT	4 capsules, Tid	CT (nitrates, lipid-lowering drugs, β-receptor blockers, enteric-coated aspirin, calcium channel blockers, ACEI, angiotensin receptor inhibitors)	8 weeks	The “Nomenclature and Diagnostic Criteria for IHD” recommended by WHO and the diagnostic criteria in “Recommendations for the Diagnosis and Treatment of UA” issued by the Cardiovascular Branch of the CMA in 2000	(6) (10) (11)
			C:67.6 ± 10						
Wu (2011)	109 (59/50)	75/34	T:66.6 ± 11.35	TXLC + CT	2 or 4 capsules, Tid	CT (clopidogrel 75 mg, Qn; LMWH calcium 5000 U, iH, Q12h, 7 days; isosorbide mononitrate, 20 mg, Bid; betaloc 12.5 mg, Bid: simvastatin, 20 mg, Qn; enalapril, 5 mg, Bid, as long as hypotension does not occur; calciumion antagonists, etc. Patients with arrhythmia, hypertension, and diabetes are given symptomatic treatments such as antihypertensive, hypoglycemic, and antiarrhythmic treatment at the same time)	10 months	“Acc/A—HA2007 UA/Non-ST-segment Elevation Myocardial Infarction Treatment Guidelines Diagnostic Criteria”	(6)
			C:63.8 ± 10.57						
Wu S. J. et al. (2006)	180 (120/60)	NR	NR	TXLC + CT	TXLC low dose group 2 capsules, Tid; TXLC high dose group 4 capsules, Tid	CT (antithrombotic, nitrate vinegar drugs, β-blockers, ACEI)	4 weeks	UA diagnostic criteria in the guidelines and recommendations for the treatment of cardiovascular diseases	(6)
Wu et al. (2010)	110 (57/53)	63/47	T:71.4 ± 4.5	TXLC + CT	4 capsules, Tid	CT (routinely give clopidogrel 75 mg/d and aspirin 100 mg/d for at least 7 days before PCI; routine treatment after PCI (such as aspirin, clopidogrel, β-blockers, nitrates, angiotensin conversion) enzyme inhibitors, LMWH, etc.)	6 months	Guidelines for the diagnosis and treatment of UA and non-ST-segment elevation myocardial infarction formulated by the Cardiovascular Branch of the CMA in 2007	(3) (4) (7)
			C:69.8 ± 4.3						
Xin et al. (2008)	128 (66/62)	73/55	T:64 ± 10	TXLC + CT	4 capsules, Tid	CT (enteric-coated aspirin, nitrate esters, β-blocker, containing nitroglycerin at the time of disease)	4 weeks	The naming and diagnostic criteria for IHD recommended by the International Society of Cardiology and WHO	(6) (8) (9)
			C:63 ± 8						
Xing (2013)	120 (60/60)	71/29	NR	TXLC + CT	4 capsules, Tid	CT (Low-fat diet, recorde resting ECG once a day; isosorbide, 10 mg, Tid; enteric-coated aspirin, 100 mg, qd; oxygen inhalation, sublingual nitroglycerin for angina pectoris, intravenous nitroglycerin, subcutaneous injection of tid LMWH, etc. if necessary)	4 weeks	WHO diagnosis and classification criteria of coronary heart disease and angina pectoris in 1979	(9)
Yang (2008)	100 (50/50)	49/51	T:62.4 ± 10.9	TXLC + CT	3 capsules, Tid	CT (nitrates, calcium antagonists, β-receptor blockers, ACEI, enteric-coated aspirin, statins)	1 month	In line with the WHO diagnosis of UA patients	(11) (17)
			C:58.2 ± 12.0						
Yang et al. (2019)	100 (50/50)	61/39	T:66.2 ± 4.8	TXLC + CT	4 capsules, Bid	CT (5-isosorbate mononitrate, 40 mg, Bid; aspirin,100 mg, Qn, before bedtime; betaloc, 25 mg, Bid)	3 months	According to the WHO diagnostic criteria for angina pectoris of coronary heart disease: typical symptoms of angina pectoris; ECG showed obvious changes of myocardial ischemia	(6) (10) (11)
			C:66.6 ± 4.7						
Yu and Chen (2015)	122 (68/54)	69/53	T:56.6 ± 3.5 C:57.2 ± 2.9	TXLC + CT	2 capsules, Tid	CT (aspirin, clopidogrel, ACEI, β-blockers, statins, nitrates, and subcutaneous injection of LMWH and other drugs)	1 year	On the basis of typical clinical manifestations, dynamic changes of ECG ST-segment elevation and depression, myocardial enzyme spectrum during angina pectoris attack, troponin was clearly diagnosed as UA patient	(3) (7)
Yu and Hu (2012)	120 (60/60)	66/54	T:55.3 ± 6.5	TXLC + CT	2 capsules, Tid	CT (betalox 50 mg, Bid; antiplatelet aggregation drugs, ACEI and lipid lowering drugs)	NR	According to the Braunwald grade, there were 46 cases in grade I, 40 cases in grade II and 34 cases in grade III.	(2) (5) (6)
			C:54.7 ± 6.2						
Yuan (2019)	100 (50/50)	53/47	T:63.14 ± 5.79	TXLC + CT	3 capsules, Qd	CT (adjusting blood glucose and controlling blood pressure; simvastatin 4 tablets/time, Qd)	4 months	The relevant diagnostic criteria for coronary heart disease UA in the Guidelines for the Diagnosis and Treatment of UA and Non-ST-Segment Elevation Myocardial Infarction formulated by the Chinese Society of Cardiology, etc.	(16)
			C:62.78 ± 5.42						
Zhang et al. (2009)	166 (86/80)	114/52	T:55	TXLC + CT	4 capsules, Tid	CT (nitrates, aspirin, β-blockers, etc.)	3 months	International Society of Cardiology and WHO Diagnostic Criteria	(6) (10)
			C:54						
Zhou (2013)	152 (78/74)	98/54	T:67	TXLC + CT	3 capsules, Tid	CT (rest on bed for 7 days, oxygen inhalation, blood pressure control; isosorbide tablets, 10 mg, Tid; atorvastatin calcium tablets, 20 mg, Qn; enteric-coated aspirin tablets, 150 mg, Qd, change to 100 mg Qd after 3 days)	1 month	The standard of the middle and high risk group for the risk stratification of UA by the Cardiovascular Branch of the CMA	(6) (10)
			C:68						

Note: CT, conventional treatment; T, trial; C, control; M, male; F, female; SD, standard deviation; TXLC, Tongxinluo capsule; CMA, Chinese Medical Association; UA, unstable angina; WHO, World Health Organization; IHD, ischemic heart disease; LMWH, low molecular weight heparin; ACEI, angiotension converting enzyme inhibitors; ECG, electrocardiogram; ASC, American Society of Cardiology; AHA, American Heart Association; CCS, Canadian Cardiovascular Society; PCI, percutaneous coronary intervention; Bid, twice a day; Tid, three times a day; Qd, once a day; iH, hypodermic injection; Qn, every night; Q12h, every 12 h. (1) Rate of cardiovascular events; (2) Mortality due to any cardiovascular event; (3) Incidence of acute myocardial infarction (AMI); (4) Revascularization (including percutaneous coronary intervention (PCI), percutaneous transluminal coronary angioplasty (PTCA) and coronary artery bypass grafting (CABG)); (5) All-cause mortality; (6) Adverse effect; (7) Recurrence of angina; (8) NST; (9) ∑ST; (10) ECG Improvement; (11) Clinical efficacy in UA; (12) Chest pain or tightness; (13) Palpitation; (14) Shortness of breath; (15) Asthenia; (16) Hypersensitive C-reactive protein (hs-CRP) Level; (17) Nitric oxide (NO) Level.

### Risk of Bias in Included Studies

**FIGURE 2:**
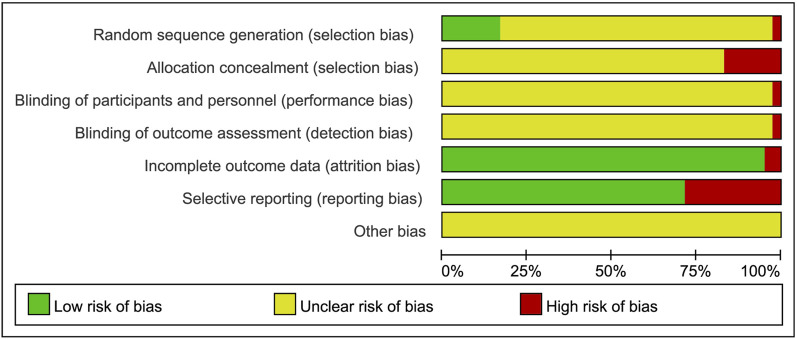
Risk of bias graph.

**FIGURE 3:**
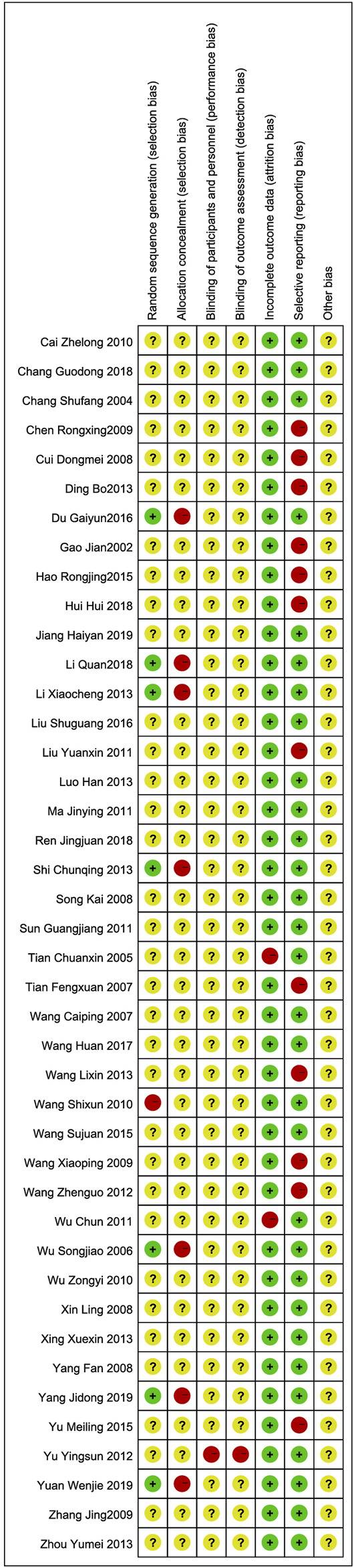
Risk of bias summary.

The qualities of 42 RCTs were evaluated from 7 aspects following the risk of bias scale in the Cochrane handbook of the Cochrane Collaboration. All selected trials reported randomized allocation of participants but rarely referred to randomization methods in sequence generation; hence, this situation was judged as unclear risk. Seven studies (Wu S. J. et al., 2006; Li., 2013; Shi., 2013; Du., 2016; Li Q. et al., 2018; Yang et al., 2019; Yuan, 2019) used random number tables to generate sequences and were rated as low risk, while the risk for allocation concealment was deemed a high level. Only 1 study (Yu and Hu., 2012) mentioned blinding without further information on specific methods, therefore other studies were considered at a high risk of bias due to unsearchable blind details. Two studies (Tian and Xu., 2005; Wu., 2011) reported 10 patient withdrawals due to the intolerance of side effects and consequently obtained a high likelihood of incomplete result. The rest of the participants finished all treatments except for 1 dropout from the control group. Selective reporting was found in 12 studies (Chen and Li, 2009; Cui, 2008; Ding et al., 2013; Gao et al., 2002; Hao., 2015; Hui et al., 2018; Liu., 2011; Tian et al., 2007; Wang et al., 2013; Wang et al., 2009; Wang et al., 2012; Yu and Chen., 2015). Beyond that, no studies mentioned attrition bias or reporting bias. Although the above information demonstrated a consistent between-group baseline, the potential sources of bias such as differences in CTs options, intent-to-treat and other adherence difference might still exist. After trying to contact the authors to clarify the unreported information *via* e-mail and receiving no reply, other biases were assessed as unclear risks. The risk of bias assessments for overall and individual studies are presented in Figures 2, 3.

### Effects of Interventions

#### Primary Outcome Measures

##### Rate of Cardiovascular Events

**FIGURE 4:**
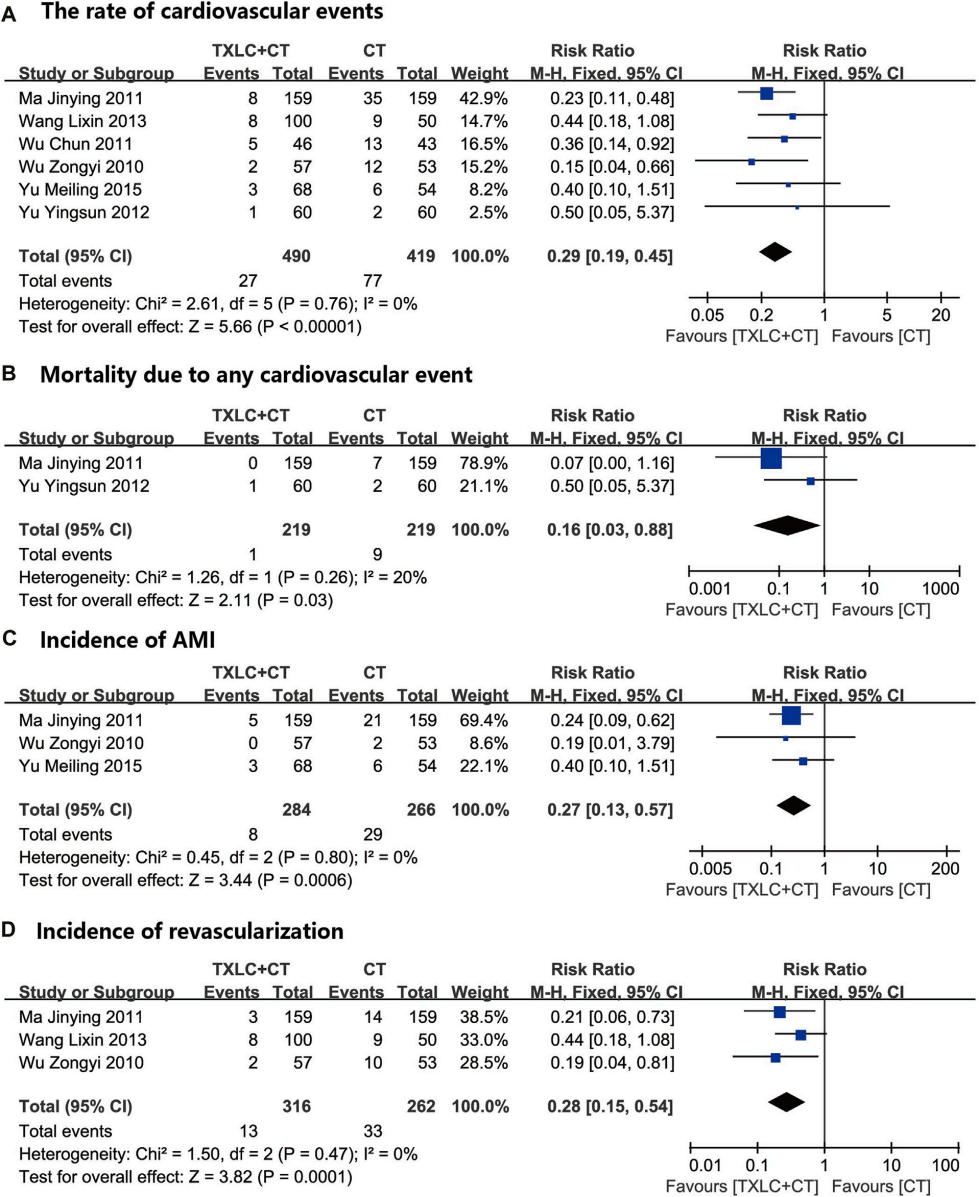
Meta-analysis of the rate of cardiovascular events (including cardiovascular mortality incidence of AMI, and incidence of revascularization) using TXLC Plus CT vs. CT.

Cardiovascular events were defined as cardiovascular death, AMI and revascularization (including PCI, PTCA, CABG). A fixed effect model was used after the heterogeneity test (*χ* ^*2*^ = 2.61, *p =* 0.76; *I* ^*2*^ = 0). Strong evidence across 909 participants in 6 studies (Wu et al., 2010; Ma et al., 2011; Wu., 2011; Yu and Hu., 2012; Wang et al., 2013; Yu and Chen., 2015) showed that taking TXLC as adjuvant therapy had a lower rate of cardiovascular events than CTs did, and a significant difference between the two groups was observed [RR = 0.29, 95% CI (0.19, 0.45), *p* < 0.00001, Figure 4A]. Except for adverse effects, the meta-analysis results of each outcome indicator is shown in Table 2.

**TABLE 2:** Summary of meta-analysis results of outcome indicators.

Outcome/indicators		No. (RCTs)	No. (participants)	MD/RR [95%CI]	*I* ^*2*^ value	Model
Rate of cardiovascular events		6	909	RR = 0.29, [0.19, 0.45]	0	Fixed
Cardiovascular events	Cardiovascular mortality	2	438	RR = 0.16, [0.03, 0.88]	20%	Fixed
	Incidence of AMI	3	550	RR = 0.27, [0.13, 0.57]	0	Fixed
	Occurrence of revascularization	3	578	RR = 0.28, [0.15,0.54]	0	Fixed
All-cause mortality		3	556	RR = 0.25, [0.06, 0.99]	19%	Fixed
Recurrence of angina		2	232	RR = 0.25, [0.11, 0.61]	0	Fixed
NST		3	350	MD = −0.45, [−0.69, −0.20]	0	Fixed
∑ST		4	470	MD = −0.70, [−1.08, −0.32]	70%	Random
ECG Improvement		13	1,640	RR = 1.23, [1.16, 1.30]	0	Fixed
Clinical efficacy in UA		19	2,342	RR = 1.26, [1.21, 1.32]	24%	Fixed
Hs-CRP Level		4	516	MD = −2.86, [−3.73, −1.99]	86%	Random
NO Level		2	208	MD = 11.67, [8.33,15.02]	33%	Fixed
Symptom improvement	Chest pain or tightness	2	220	RR = 1.13, [0.97, 1.32]	30%	Fixed
	Palpitation	2	191	RR = 1.47, [1.18,1.84]	0	Fixed
	Shortness of breath	2	193	RR = 1.53, [1.24,1.88]	0	Fixed
	Asthenia	2	221	RR = 1.69, [0.83, 3.43]	90%	Random

Note: AMI, acute myocardial infarction; CI, confidence interval; MD, mean difference; NST, number of ST-segment depression; RCT, randomized controlled trial; RR, risk ratio; UA, unstable angina.

Two trials (Ma et al., 2011; Yu and Hu., 2012) with a total of 438 participants compared the efficacy of two interventions on cardiovascular mortality. The merged result indicated that TXLC combined with CT showed a better potential for reducing cardiovascular mortality which with low heterogeneity [*χ* ^2^ = 1.26, *p =* 0.26; *I* ^*2*^ = 20%; RR = 0.16, 95% CI (0.03, 0.88), *p =* 0.03, Figure 4B].

The incidence of AMI was evaluated in 550 participants of 3 trials (Wu et al., 2010; Ma et al., 2011; Yu and Chen., 2015). The meta-analysis result showed that adding TXLC to CT reduced the onset of AMI [RR = 0.27, 95% CI (0.13, 0.57), *p =* 0.0006], and no statistical heterogeneity was detected (*χ* ^2^ = 0.45, *p* = 0.80; *I* ^*2*^ = 0, Figure 4C).

Three trials (Wu et al., 2010; Ma et al., 2011; Wang et al., 2013) with 578 participants reported the occurrence of revascularization. Among them, Ma et al. (2011) observed the implementation of PTCA/CABG, Wang et al. (2013) did not mention the type of emergency revascularization, and Wu et al. (2010) recorded the patients who received PCI. Meta-analysis demonstrated a lower incidence of revascularization in the trial group [*χ* ^2^ = 1.50, *p* = 0.47; *I* ^*2*^ = 0, RR = 0.28, 95% CI (0.15, 0.54), *p =* 0.0001, Figure 4D].

##### All-Cause Mortality

**FIGURE 5:**
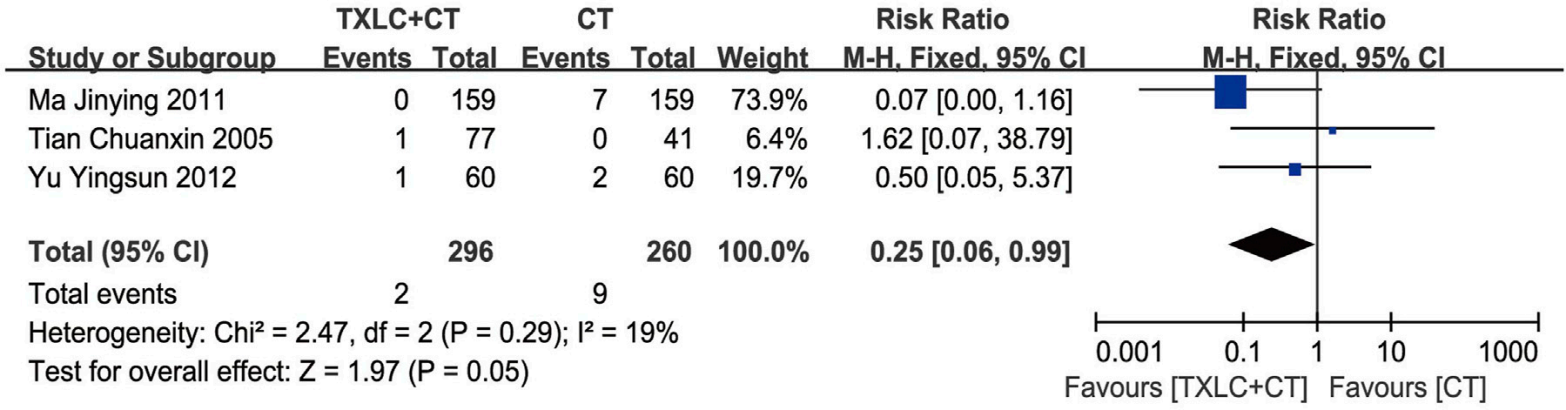
Meta-analysis of the all-cause mortality using TXLC Plus CT vs. CT.

Three trials (Tian and Xu., 2005; Ma et al., 2011; Yu and Hu., 2012) involving 556 participants reported the all-cause mortality in both trial and control groups. In consideration of the low heterogeneity (*χ* ^2^ = 2.47, *p =* 0.29; *I* ^2^ = 19%), we performed a fixed effect model for the meta-analysis. The pooled result showed that the all-cause mortality in the trial group was significantly lower than that in the control group [RR = 0.25, 95% CI (0.06, 0.99), *p =* 0.05, Figure 5].

##### Adverse Effect

Twenty-two studies mentioned adverse reactions during therapy (Tian and Xu., 2005; Wu S. J. et al., 2006; Tian et al., 2007; Wang and Li., 2007; Cui., 2008; Song et al., 2008; Xin et al., 2008; Chen and Li., 2009; Wang et al., 2009; Zhang and Li., 2009; Cai and Li., 2010; Ma et al., 2011; Wu., 2011; Wang et al., 2012; Yu and Hu., 2012; Shi., 2013; Zhou., 2013; Du., 2016; Liu and An, 2016; Wang., 2017; Ren et al., 2018; Yang et al., 2019), and 12 of them presented with adverse effects from 12 trial groups and 2 control groups. Two trials (Wang and Li., 2007; Ma et al., 2011) recorded 1 gingival bleeding case from the trial group; comparatively, no case of bleeding gums was found in the control group. One study (Yang et al., 2019) found 3 cases of hypotension from both trial and control groups, and only 1 case used TXLC as an auxiliary treatment. They also observed 1 case of bradycardia in both groups.

A total of 11 trials reported 41 patients treated with TXLC as an auxiliary therapy who showed gastrointestinal symptoms (Tian and Xu., 2005; Wu S. J. et al., 2006; Wang and Li., 2007; Cui., 2008; Xin et al., 2008; Wang et al., 2009; Ma et al., 2011; Wu., 2011; Zhou., 2013; Du., 2016; Liu and An., 2016), mainly manifested as bloating, belching, nausea, loss of appetite, acid reflux or dull pain, while only 5 participants from the control group in 1 trial experienced the same discomforts (Liu and An, 2016). Two trials (Tian and Xu., 2005; Wu., 2011) recorded 9 TXLC supplementary cases that stopped the trial due to gastrointestinal reactions. One case (Du., 2016) with TXLC had mild discomfort in the upper abdomen, and symptoms disappeared after being given gastric mucosal protective agents. Two trials (Cui., 2008; Wang et al., 2009) reported 5 cases of epigastric discomfort, acid reflux, nausea or dull pain, and symptoms disappeared when TXLC was taken after meals. A trial (Zhou., 2013) reported that the participants experienced epigastric discomforts after TXLC and aspirin were treated combinedly, which disappeared when treated separatedly at an hour’s interval.

There were no withdrawals from the trials due to hypotension, bradycardia or gum bleeding. No other adverse effect was reported. In summary, it is premature to conclude that TXLC is safe based on existing data. The details of adverse effects are shown in Table 3.

**TABLE 3:** The incidences of main adverse effects and of TXLC Plus CTs *vs.* CTs

Adverse effects	TXLC plus CTs		Studies	CTs		Studies
	Adverse effect (n)	Trials(n)		Adverse effect (n)	Trials(n)	
Gastrointestinal symptoms such as bloating, belching, nausea, loss of appetite, acid reflux, and dull pain	41	11	Cui (2008), Du (2016), Liu and An (2016), Ma et al. (2011), Tian and Xu (2005), Wang and Li (2007), Wang et al. (2009), Wu (2011), Wu S. J. et al. (2006), Xin et al. (2008), Zhou (2013)	5	1	Liu and An (2016)
Hypotension	1	1	Yang et al. (2019)	2	1	Yang et al. (2019)
Bleeding gums	2	2	Ma et al. (2011), Wang and Li (2007)	0	0	no
Bradycardia	1	1	Yang et al. (2019)	1	1	Yang et al. (2019)

Note: TXLC, Tongxinluo capsule; CTs, conventional treatments.

#### Secondary Outcomes

##### Recurrence of Angina

**FIGURE 6:**
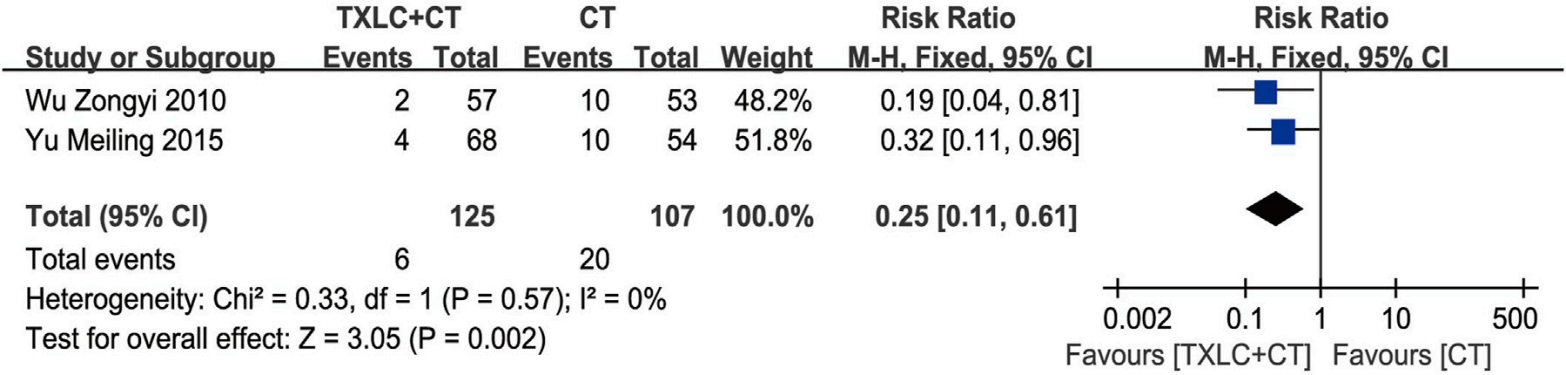
Meta-analysis of recurrence of angina using TXLC Plus CT vs. CT.

Two included (Wu et al., 2010; Yu and Chen., 2015) trials reported recurrences of angina. A fixed effect model was performed due to zero between-trial heterogeneity (*χ* ^*2*^ = 0.33, *p* = 0.57; *I* ^2^ = 0%). A total of 232 participants were followed for 6 months and angina frequency was recorded during this period. The forest plot showed that adding TXLC to CTs reduced the recurrence of angina pectoris. There was a significant difference between the 2 groups [RR = 0.25, 95% CI (0.11, 0.61), *p =* 0.002, Figure 6].

##### NST

**FIGURE 7:**
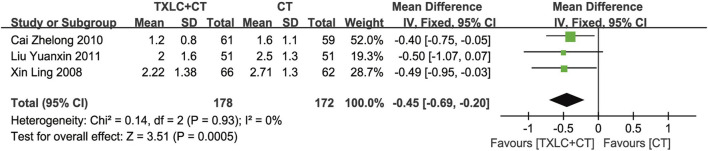
Meta-analysis of NST using TXLC Plus CT vs. CT.

Three trials (Xin et al., 2008; Cai and Li., 2010; Liu., 2011) measured the number of ST-segment depressions in UA patients. After testing heterogeneity (*χ* ^*2*^ = 0.14, *p* = 0.93; *I* ^*2*^ = 0%), a fixed effect model was used. Under the 2 different treatments, the depression number in the trial group was less than that in the control group among 350 participants, indicating that TXLC improved the ECG characterization of myocardial ischemia [MD = −0.45, 95% CI (−0.69, −0.20), *p =* 0.0005, Figure 7].

##### ∑ST

**FIGURE 8:**
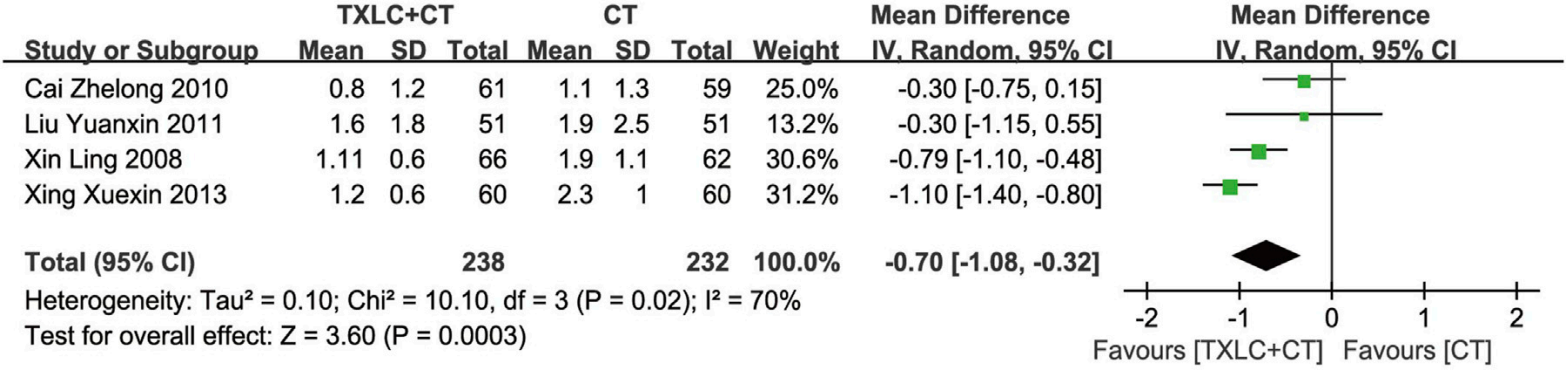
Meta-analysis of ∑ST using TXLC Plus CT vs. CT.

The summation of ST-segment depression reflects the degree of myocardial ischemia. It was evaluated in 470 participants from 4 trials (Xin et al., 2008; Cai and Li., 2010; Liu., 2011; Xing., 2013). The heterogeneity was more than 50% (*χ* ^*2*^ = 10.10, *p =* 0.02; *I* ^2^ = 70%), and a random effect model was chosen. Meta-analysis showed that the total declines of ST-segment in the trial group was lower than the control group, and the difference between the 2 groups was statistically significant [MD = −0.70, 95% CI (−1.08, −0.32), *p =* 0.0003, Figure 8].

##### ECG Improvement

**FIGURE 9:**
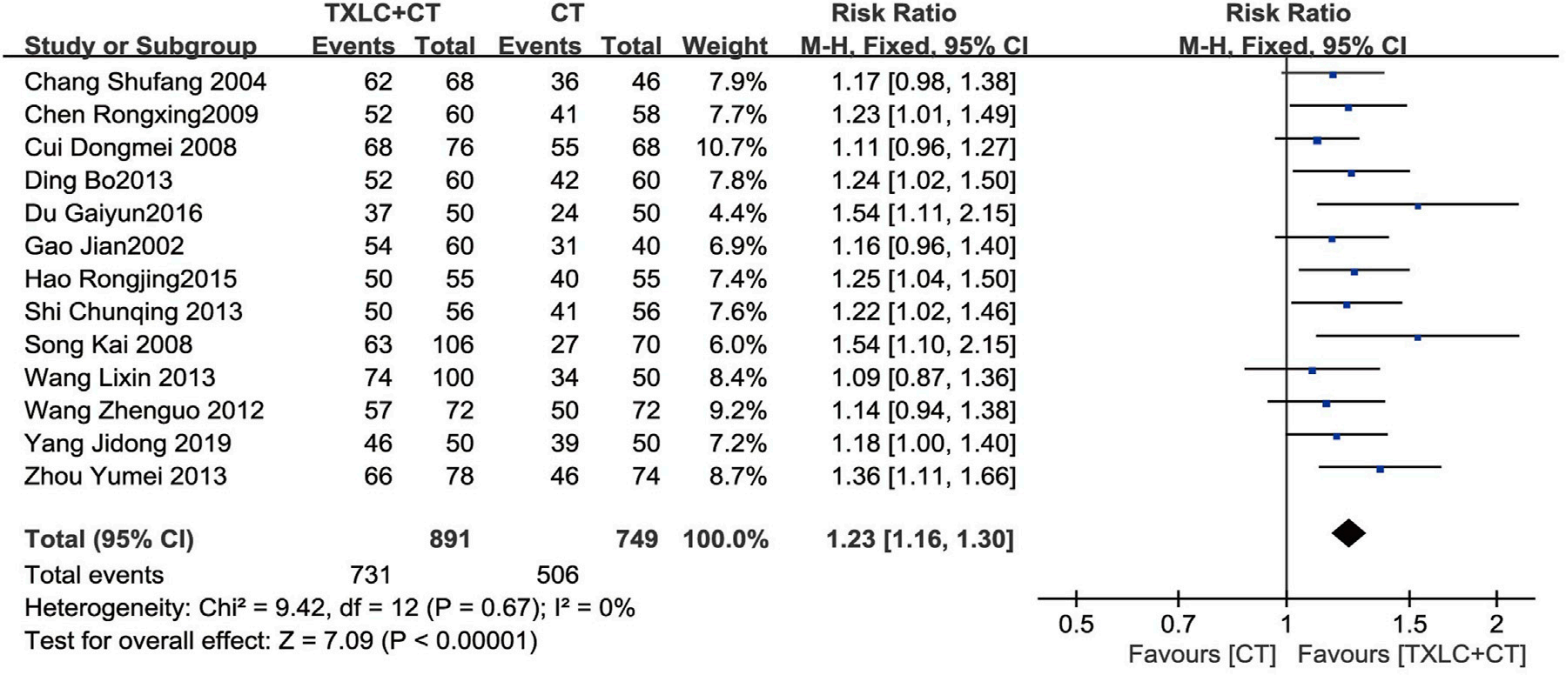
Meta-analysis of ECG improvement using TXLC Plus CT vs. CT.

Thirteen studies (Chang and Zhao, 2004; Chen and Li, 2009; Cui., 2008; Ding et al., 2013; Du., 2016; Gao et al., 2002; Hao., 2015; Shi., 2013; Song et al., 2008; Wang et al., 2013; Wang et al.,2012; Yang et al., 2019; Zhou., 2013) mentioned ECG improvement. Using a fixed effect model was reasonable owing to nonexistent between-trial heterogeneity (*χ* ^*2*^ = 9.42, *p =* 0.67; *I* ^2^ = 0%). The meta-analysis result showed that combined with CTs, TXLC showed an improvement in the effectiveness of the ECG. There was a statistically significant difference between the 2 groups [RR = 1.23, 95% CI (1.16, 1.30), *p* < 0.00001, Figure 9].

##### Clinical Efficacy in UA

**FIGURE 10:**
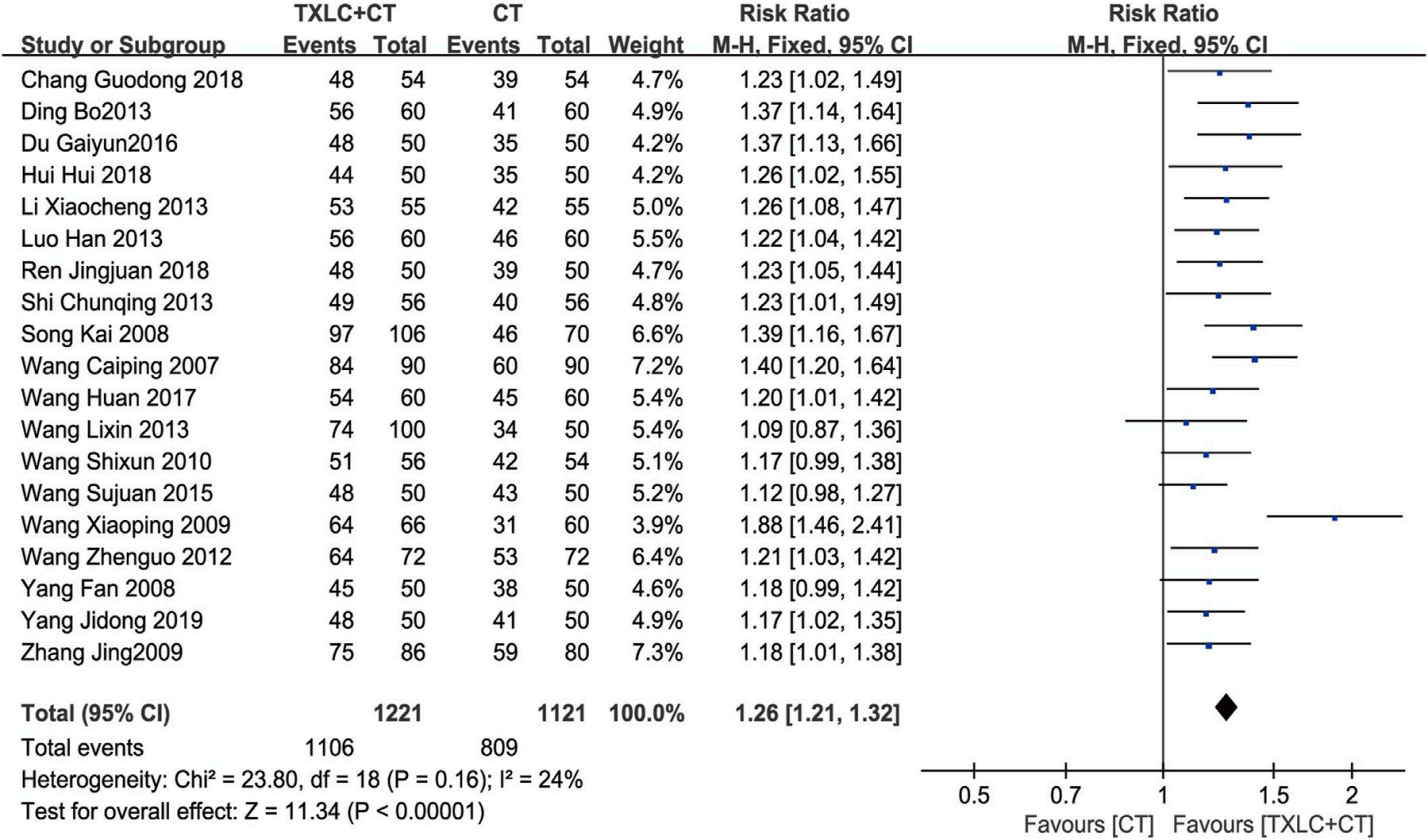
Meta-analysis of the clinical efficacy in UA using TXLC Plus CT vs. CT.

Nineteen RCTs (Wang and Li., 2007; Song et al., 2008; Yang., 2008; Wang et al., 2009; Zhang and Li 2009; Wang et al., 2010; Wang et al., 2012; Ding et al., 2013; Li., 2013; Luo., 2013; Shi., 2013; Wang et al., 2013; Wang., 2015; Du., 2016; Wang., 2017; Chang et al., 2018; Hui et al., 2018; Ren et al., 2018; Yang et al., 2019) reported the clinical efficacy for UA. A fixed effect model was adopted for merging data after testing heterogeneity (*χ* ^*2*^ = 23.80, *p =* 0.16; *I* ^2^ = 24%). The merged results suggested that TXLC combined with CTs was better than CTs alone in improving the clinical efficacy of angina pectoris. A significant difference was observed between groups [RR = 1.26, 95% CI (1.21, 1.32), *p* < 0.00001, Figure 10].

##### Symptom Improvement

**FIGURE 11:**
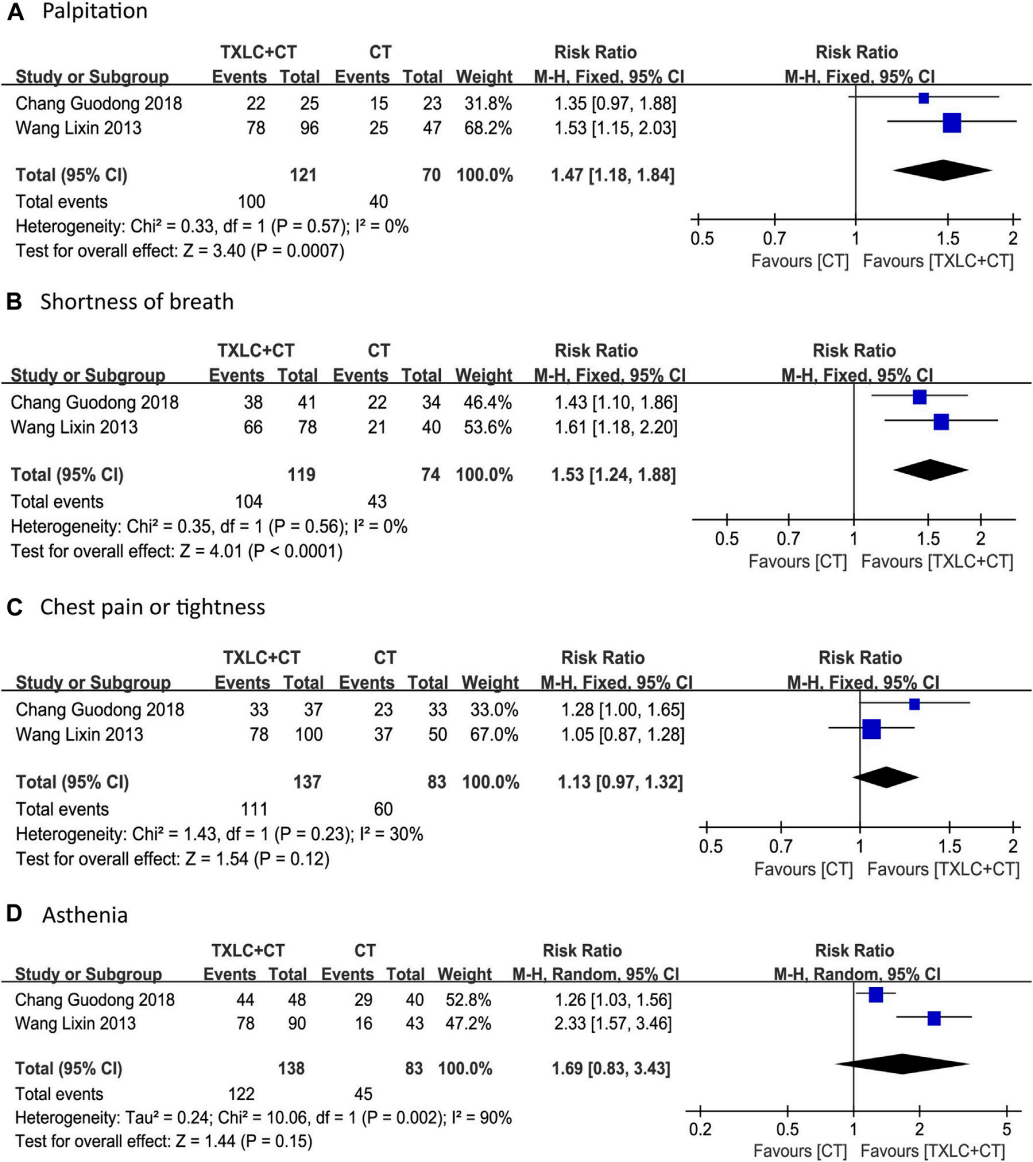
Meta-analysis of symptom improvement (including chest pain or tightness, palpitation, shortness of breath, asthenia) using TXLC Plus CT vs. CT.

Two studies (Wang et al., 2013; Chang et al., 2018) compared symptom improvement. Unlike the clinical efficacy for UA, which has specific diagnostic criteria and consensus, symptom improvement includes remissions of chest pain or tightness, palpitation, shortness of breath and asthenia. In contrast with these quantifiable indicators, such as ECG, consumption of nitroglycerine, and frequency and duration of angina attack, the symptoms here emphasized the patient’s overall disease state. Because low between-trial heterogeneity for chest pain or tightness (*χ* ^*2*^ = 1.43, *p =* 0.23; *I* ^2^ = 30%) was shown, and no heterogeneity for palpitation (*χ* ^*2*^ = 0.33, *p =* 0.57; *I* ^2^ = 0%) or shortness of breath (*χ* ^*2*^ = 0.35, *p =* 0.56; *I* ^2^ = 0%) were found, fixed effect models were selected. However, a random effect model was used for asthenia due to its high heterogeneity (*χ* ^*2*^ = 10.06, *p =* 0.002; *I* ^2^ = 90%). The meta-analyses indicated that the trial group had more remissions of palpitation [RR = 1.47, 95% CI (1.18, 1.84), *p =* 0.0007, Figure 11A] and shortness of breath [RR = 1.53, 95% CI (1.24, 1.88), *p* < 0.0001, Figure 11B], the difference between groups was statistically significant. Further, the trial group showed an improvement of chest pain or tightness and asthenia, but there was no significant difference compared with the control group [RR = 1.13, 95% CI (0.97, 1.32), *p =* 0.12, Figure 11C; RR = 1.69, 95% CI (0.83, 3.43), *p* = 0.15, Figure 11D].

##### Hs-CRP Level

**FIGURE 12:**
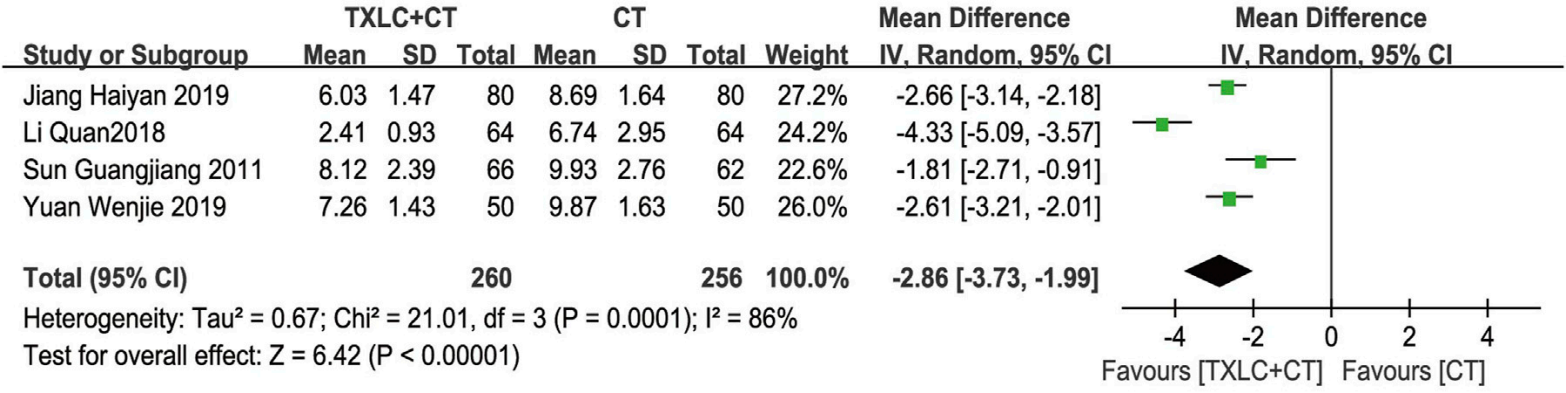
Meta-analysis of hs-CRP level using TXLC Plus CT vs. CT.

Hs-CRP was evaluated in a total of 4 studies (Sun et al., 2011; Li Q. et al., 2018; Jiang et al., 2019; Yuan., 2019). As high between-trial heterogeneity was shown (*χ* ^*2*^ = 21.01, *p =* 0.0001; *I* ^2^ = 86%), a random effect model was performed. Meta-analysis showed that conventional drugs combined with TXLC significantly reduced serum hs-CRP [MD = -2.86, 95% CI (−3.73, −1.99), *p* < 0.00001, Figure 12].

##### NO Level

**FIGURE 13:**
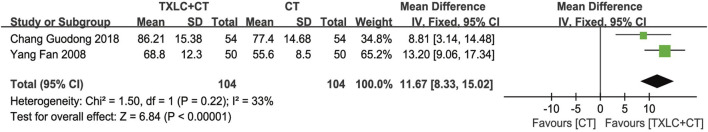
Meta-analysis of NO level using TXLC Plus CT vs. CT.

Evidence from pooled analysis across two studies (Yang., 2008; Chang et al., 2018) showed that TXLC plus CTs produced greater increase of the plasma level of NO over CTs [MD = 11.67, 95% CI (8.33, 15.02), *p* < 0.00001, Figure 13] with no significant heterogeneity (*χ* ^*2*^ = 1.50, *p =* 0.22; *I* ^2^ = 33%).

### Sensitivity Analysis

When discussing hs-CRP and ∑ST, the analysis results showed high between-trial heterogeneities, so sensitivity analyses were implemented by excluding each study. After inspecting, the heterogeneity of hs-CRP decreased from *I* ^*2*^ = 86% to *I* ^2^ = 29% after excluding Li Q. et al. (2018). When Xing (2013) was excluded in ∑ST, the heterogeneity decreased from *I* ^*2*^ = 70% to *I* ^2^ = 45%. Both indicators’ heterogeneities were reduced from a high level to less than 50%, indicating that the results of hs-CRP and ∑ST were not stable enough.

After rechecking the data, the possible sources of heterogeneities of the two indicators were discovered. The serum hs-CRP concentration before treatment recorded by Li Q. et al. (2018) was not significantly different from the other three studies. After treatment, the average hs-CRP concentration of the two groups was lower than other included trials, the average concentration of the trial group even dropped to 2.41 mg/L. After excluding the influence of the patients’ baseline and medication course, three possible sources of heterogeneity were inferred: 1) The total daily dose of TXLC in Li Q. et al. (2018) was 6, which was the lowest of the three groups; 2) Specific CTs programs were not mentioned, and there were differences in the version and content of the diagnostic criteria between the 4 RCTs; 3) Errors occurred in the process of testing and data recording. It is known that the hs-CRP level in patients with coronary heart disease is relatively high, while the average value of the Li Q. et al. (2018) treatment group was very close to the normal standard. As the authors of the original article were uncontactable, the main source of the heterogeneity has not yet been determined. Regarding ∑ST, no obvious source of heterogeneity was found except for the differences in the UA diagnostic criteria of the 4 RCTs. The forest plot was shown in Supplementary Material S4.

### Publication Bias

**FIGURE 14:**
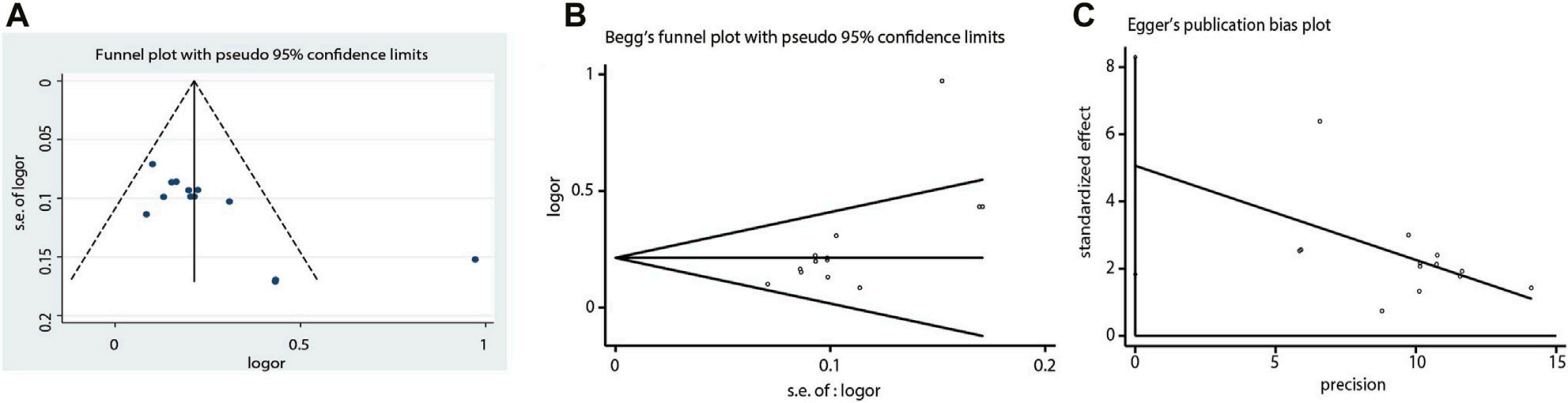
Funnel plot, Begg’s funnel plot and Egger’s publication bias plot of the ECG improvement.

**FIGURE 15:**
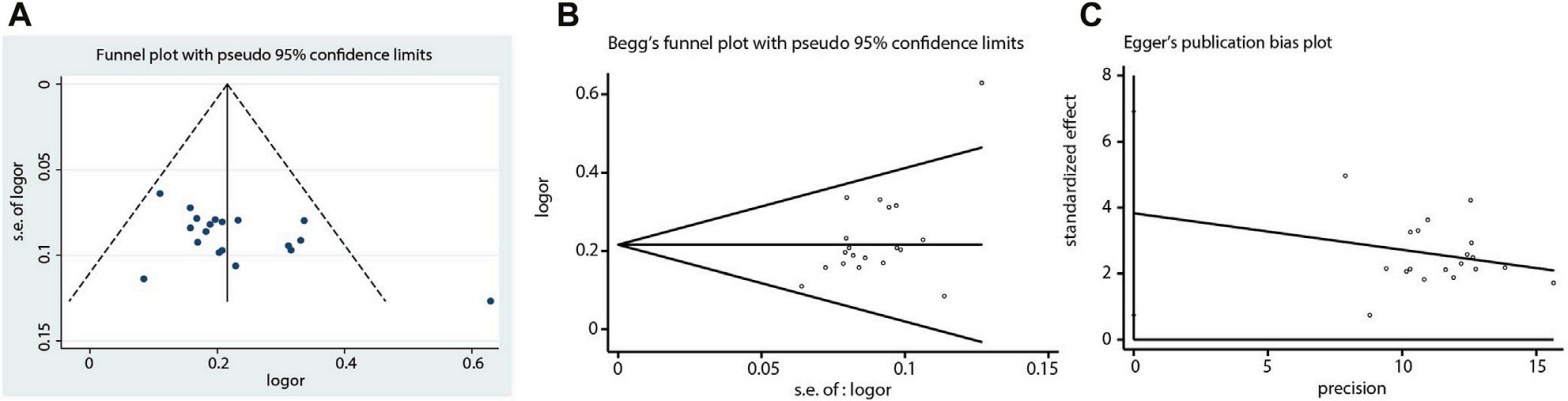
Funnel plot, Begg’s funnel plot and Egger’s publication bias plot of the clinical efficacy in UA.

The number of RCTs included in the ECG improvement and clinical efficacy in UA were 13 and 19, which were greater than 10, so funnel plots were constructed to assess potential publication bias. Since funnel plots of both indicators showed slight asymmetries in the scatter distribution, it was considered that certain degree of publication biases might exist. This conclusion was consistent with the Begg (ECG improvement: Z = 2.01, *p =* 0.044; angina pectoris efficacy: Z = 1.61, *p =* 0.108) and Egger tests (ECG improvement: t = 3.45, *p =* 0.005; angina pectoris efficacy: t = 2.62, *p =* 0.018), which indicated that there should be publication biases to a certain extent. Factors such as insufficient sample sizes and the lack of reporting on negative results were the possible causes of publication biases (Figures 14A–C, Figures15A–C).

## Discussion

### Summary of Main Results (Benefits and Harms)

A total of 5421 UA patients in 42 RCTs were included in this systematic review. Meta-analysis showed that TXLC, as an adjunct to CT, reduced the incidence of cardiovascular events and all-cause mortality, improved ECG performance, and relieved angina symptoms and other accompanying symptoms. It also showed beneficial effects on regulating serum hs-CRP level and plasma NO concentration. Oral TXLC medication caused few adverse effects with mild symptoms, which were mostly eliminated by adjusting the medication course, reducing the dose, or giving symptom-relieving drugs. Few patients chose to withdraw from the clinical trials on account of unbearable adverse effects. Among all the results, only the concentrations of hs-CRP (*I* ^*2*^ = 86%) and ∑ST (*I* ^*2*^ = 70%) demonstrated high heterogeneities. Except for the improvement of chest pain and tightness and asthenia, all intergroup differences of the remaining results showed statistical significance.

Meta-analysis revealed that TXLC was beneficial to reducing UA endpoint events. The general incidence of cardiovascular events in the trial group was lower than that in the control group without between-trial heterogeneity, and the result manifested good stability in the sensitivity analysis. It is noteworthy that except for two studies (Ma et al., 2011; Yu and Hu., 2012) not recording the duration of treatment, the follow-up times of the other 4 studies (Wu et al., 2010; Wu., 2011; Wang et al., 2013; Yu and Chen., 2015) were 6 months, 10 months, 4 weeks and 1 year. Further comparison showed that the endpoint event rate of UA patients with a 1 year course of treatment was 3.3%, which was the lowest among the 4 trials, and notably, the trial with 6 months course of treatment had 5.5 times end point event rate in the control group higher than that of the trial group (Wu et al., 2010). Based on this, it was speculated that TXLC had the potential to reduce endpoint events in both long-term and short-term adjuvant treatments. In this meta-analysis, 6 months and above TXLC treatment showed better effect in reducing cardiovascular events. However, under the limitation of incomplete data, it was hard to fully conclude that the curative effect was proportional to the treatment time. When analyzing all-cause mortality and cardiovascular mortality, two analogical results appeared. After comparing all included RCTs, it was found that one RCT reported an accidental death case in the trial group, which was the only difference between all cases of the two indicators. Thus, no definitive conclusions could be drawn on TXLC reducing all-cause mortality of UA when it worked as an adjunct to CTs.

Additionally, TXLC had a good performance in reducing UA recurrence, improving ECG parameters and alleviating angina symptoms. Two RCTs showed that the rate of UA recurrence in the trial group was only 25% of that in the control group, suggesting that TXLC might better prevent UA recurrence. In terms of ECG parameters, TXLC was found to significantly reduced the degree of myocardial ischemia in UA by reducing the ST-segment depression number and the total depression distance with an average of 0.45 and 0.70 mm, respectively. The ECG improvement was defined as an elevation of the ST-segment over 0.05 millivolt. Among 13 RCTs, 731 (82.0%) of 891 patients undergoing TXLC treatment showed improvement in ECG, while 506 (67.6%) of 749 patients treated with conventional drugs exhibited effective responses, indicating striking differences in efficacy between groups. As the clinical efficacy in UA was the most frequently reported indicator, the results showed that 1,106 out of 1,221 patients treated with TXLC demonstrated reduced symptoms of angina, which reached 90.58% effective rate and better than 72.17% in the control group. It was also found that taking TXLC based on CTs significantly improved a series of symptoms of UA including chest pain or tightness, palpitations, shortness of breath, and asthenia. However the improvement of chest pain or tightness and asthenia presented a high degree of heterogeneity, which might attribute to the inconsistency of the baseline. Based on the above analysis, TXLC showed improvement on the overall efficacy as an auxiliary treatment, which might rely on its multitarget and multichannel mode of action (Wang et al., 2021).

Another valuable finding was the effects of TXLC on regualating hs-CRP and NO levels. As an acute-phase protein synthesized under an inflammatory state, hs-CRP is of diagnostic and prognostic value for acute coronary syndrome. In this study, the trial group supplemented with TXLC reduced the serum hs-CRP level in UA patients by 2.86 mg/L compared with that of the control group. However, the results showed high heterogeneity ascribed to low trial quality and unmeasured hs-CRP baselines. Nitrates are known to exert their vasodilation and anti-angina effects by converting to NO in the body, and traditional Chinese medicine may regulate NO concentration through multiple pathways. Experimental evidence has shown that TXLC regulates NO synthesis by changing the activity of endothelial NO synthase, thereby protecting the myocardium from ischemia/reperfusion injury. Two RCTs included in this study recorded plasma NO levels. Compared with the control group, the plasma NO level of the trial group increased 11.67 μmol/L, showing that TXLC could regulate NO synthesis, which related to its effects of promoting vascular endothelial repair, improving endothelial cell function, reducing vascular tone and ameliorating coronary blood supply (Liu et al., 1996).

Safety of TXLC is of great concern because of its wide application. No serious adverse effect were mentioned in the included studies, while 4 adverse effects were described in 22 RCTs, including gastrointestinal reactions, hypotension, gum bleeding and palpitation. Gastrointestinal discomfort was the most frequently reported adverse effect. Eleven RCTs reported gastrointestinal reactions in the trial group, and the treatment was interrupted in 9 patients in 2 trials (Yang., 2008; Wang et al., 2010) due to intolerance. Even though most of the side effects could be eliminated or alleviated through dose reduction, medication time adjustment and symptomatic remedy, the non-negligible proportion of the patients who discontinued treatment due to gastrointestinal reactions (9/41) deserve further attention. In addition, 1 RCT reported hypotension and bradycardia in both trial and control groups and there was no statistically significant difference in the total incidence of adverse effects between the two groups (Yang et al., 2019). Owing to the differences in age, gender, course of the disease, comorbidities in UA and the CT regimens among the included studies, the correlation between these factors and the incidence of adverse effects was analysized. But apart from the regimen of CTs, no direct relationship between other factors and adverse effects has been found. It is worth noting that in 11 studies with gastrointestinal symptoms, aspirin was used as CTs in 8 studies (Tian and Xu., 2005; Wang and Li., 2007; Cui., 2008; Xin et al., 2008; Ma et al., 2011; Zhou., 2013; Du., 2016; Liu and An., 2016), clopidogrel was used as CT in 1 study (Wu., 2011), atorvastatin, simvastatin or other lipid-lowering drugs was used as CTs in another 5 studies (Tian and Xu., 2005; Cui., 2008; Wu., 2011; Zhou., 2013; Liu and An., 2016). Although gastrointestinal discomforts are the side effects of anticoagulants and lipid-lowering drugs (Sugisaki et al., 2018; Zhao, 2020), only 1 of the 11 RCTs (Liu and An., 2016) reported gastrointestinal discomforts in the control group, indicating the occurrence of gastrointestinal discomforts in the trial group might not be attributed to the CTs intervention. Similarly, bleeding, another side effects in UA treatment, did not reported in the control group of the included studies. In summary, the difference in CTs composition might not be a source of bias on adverse effects for this meta-analysis.

### Consistency and Disagreement with Other Researches or Reviews

Three previous meta-analyses (one in English and 2 in Chinese) of TXLC for UA patients were retrieved (Wu T. et al., 2006; Wu et al., 2018; Yang et al., 2021). After comparing the previous work with the present one vertically and horizontally, all studies indicated that UA patients treated with TXLC as an auxiliary therapy had better clinical outcomes on angina and ECG than those treated with CTs only, and most of the results showed low heterogeneity despite the low quality of the studies included.

The difference between the 4 meta-analyses was first reflected in the changes in the number, quality, and outcome indicators of the included studies. The meta-analysis published in the Cochrane Library in 2006 compared the incidence of cardiovascular events, sudden death, and angina pectoris scores between TXLC and CTs for the first time (Wu T. et al., 2006). The analysis showed TXLC had no advantages over conventional drugs in reducing the incidence of cardiovascular events, decreasing the risk of sudden death, or improving the angina pectoris score, which were different from the positive results in the present study. These inconsistencies might be partially due to the small sample size, high heterogeneity, and the limited number of included studies. This meta-analysis also reported that TXLC alleviated the onset of acute angina pectoris, and reduced the consumption of nitroglycerin as well despite the high heterogeneity (Wu T. et al., 2006). Besides, both TXLC and isosorbide mononitrate showed a reduced effect on endothelin level, and no significant quantitative difference was found between them (Wu T. et al., 2006). The meta-analysis published in 2018 only reported the efficacies of TXLC on angina pectoris and ECG, but included more high-quality studies compared with the previous one (Wu T. et al., 2006; Wu et al., 2018). Both meta-analyses confirmed that the combination of TXLC and CTs showed better effects than CTs alone on reducing the degree of angina and improving ECG. In 2020, a meta-analysis further expanded the number of included studies (Yang et al., 2021), which included indicators of hs-CRP, vascular endothelial cytokines, blood lipids and hemodynamics, indicating that TXLC might play a macroscopic role in treating UA through mechanisms of anti-inflammatory, anticoagulant, antioxidant, or endothelial protection. Today, based on a large amount of clinical data, this article investigated TXLC’s impact on UA endpoint events, and comprehensively summarized its adverse effects and the corresponding mitigation methods as well as the effects of TXLC on the recurrence of angina pectoris after recovery, the improvement of the overall symptoms, and NO level. The results confirmed that TXLC could reduce the occurrence of UA endpoint events and angina recurrence after recovery, improve the symptoms of UA, and increase the level of serum NO. Compared with 3 previous meta-analyses, this meta-analysis not only overturned the previous conclusion that TXLC was not effective for UA cardiovascular events, but also indicated the improvement of TXLC on angina pectoris sypmtoms, ECG, hs-CRP level, etc. New indicators were also observed, for example, symptoms of UA, providing additional evidence-based medicine data support for TXLC in the treatment of UA. However, a sample size of no less than 100 was set in this study in order to improve the accuracy of estimates. As a result, many of the indicators in the unqualified study were not selected, possibly leading to the limitations in this research.

Another rule found along the timeline was the change in follow-up times. In the meta-analysis of 2006, 15 of 18 studies on TXLC had a follow-up time no longer than 4 weeks, while the longest follow-up time was 8 weeks reported in 2018, which reached to 24 weeks in 2020. In the present study, the follow-up time was extended to as long as 10 months or even to 1 year. Therefore, although it still needs improvements on the scale and quality of long-term follow-up studies, the present work does contribute to the conclusion of long-term effects of TXLC intervention for UA. But it should not be neglected that the quality of RCTs in the above-mentioned 3 meta-analyses was an inescapable key weakness (Wu T. et al., 2006; Wu et al., 2018; Yang et al., 2021), which might directly affect the reliability of evidence-based medicine. High-quality and large-scale trials are essential for obtaining mature and stable evidence-based conclusions which will provide better guidance for clinical practice.

### Limitations

1) Comprehensive searches was conducted in the designated database without restricting language, ethnicity, or literature type. Since proprietary Chinese medicines have not been promoted globally, all participants ultimately included were Chinese due to the limitation of application scope.
2) The sample size was strictly limited to no less than 100 people, which led to the abandonment of some observation indicators included in small-sample studies, and several preset outcome indicators were finally discarded for the absence of the corresponding research.
3) Among the 42 studies included, only 7 of them used the random sequence generation method with high allocation concealment risk, and 1 reported the use of blinding. The related authors were contacted for details of allocation concealment and randomization methods, but no receivedwas responsed.
4) All participants had complete general information, and the between-group difference at baseline was not statistically significant. However, the difference in follow-up times and conventional prescriptions was likely to be one of the heterogeneity sources.
5) The patients from some of the included trials only received single-agent therapy which did not conform to standard treatment protocols (Wang et al., 2009; Li Q. et al., 2018). This might affect the efficacy of TXLC and lead to false-positive results.
6) Among all trials included, most of the follow-up times were within 3 months, and the longest one was only 1 year (Yu and Chen., 2015). The long-term benefit of TXLC for UA patients cannot be scaled.

## Conclusion

Taken together, this meta-analysis showed that TXLC could reduce the rate of cardiovascular, all-cause mortality and the number and summation of ST-segment depression, decreased serum hs-CRP level, improved the ECG abnormalities and clinical efficacy in UA, relieved the UA symptoms, as well as increased plasma NO concentrations. As an adjunctive treatment for UA, TXLC had a wide range of clinical effects, remarkable efficacy and good stability. Nevertheless, even if no serious adverse effects have been found, discomforts such as gastrointestinal symptoms, bleeding gums, bradycardia, and hypotension still occurred at an inconvenient low frequency. Therefore, no definitive conclusions can be drawn on its absolute safety so far, and medical staff should pay close attention to its administration.

Currently, the clinical efficacy of TXLC for UA is mainly validated *via* randomized or semi-randomized controlled trials. Inherent problems such as the high risk of bias, low quality of evidence and small samples are likely to exist. The insufficient evidence in the clinical studies of TXLC can be ameliorated by expanding the sample size and carrying out multicenter studies. It is hoped the remedies for poor quality evidence will be found early, and that more attention will be paid to the quality of life, compliance and cost acceptance in future clinical trials.

## Data Availability

The original contributions presented in the study are included in the article/Supplementary Material, further inquiries can be directed to the corresponding authors.
