# Supplementary material for: Efficacy and Safety of Tongxinluo Capsule as Adjunctive Treatment for Unstable Angina Pectoris: A Systematic Review and Meta-Analysis of Randomized Controlled Trials
Source: Front Pharmacol. 2021 Oct 11;12:742978. doi: 10.3389/fphar.2021.742978 (PMC8544810; doi:10.3389/fphar.2021.742978)
Supplement: Supplementary file 1 [file DataSheet1.zip › Supplementary Material 2. Search strategies.docx]

***Supplementary Material 1.***

The detailed search strategies

**1.Cochrane Central Register of Controlled Trials (CENTRAL)**

Search Name: tongxinluo

Date Run: 31/08/2021 12:05:37

Comment: UA

ID Search Hits

#1 MeSH descriptor: [Angina, Unstable] explode all trees 1144

#2 (Anginas, Unstable): ti,ab,kw OR (Unstable Anginas):ti,ab,kw OR (Angina Pectoris, Unstable):ti,ab,kw OR (Angina Pectori, Unstable):ti,ab,kw OR (Unstable Angina Pectori):ti,ab,kw 2340

#3 (Unstable Angina Pectoris): ti,ab,kw OR (Unstable Angina):ti,ab,kw OR (Angina at Rest):ti,ab,kw OR (Angina, Preinfarction):ti,ab,kw OR (Anginas, Preinfarction):ti,ab,kw

5140

#4 (Preinfarction Angina): ti,ab,kw OR (Preinfarction Anginas):ti,ab,kw OR ("myocardial preinfarction syndrome"): ti,ab,kw OR (Myocardial Preinfarction Syndromes):ti,ab,kw OR (Preinfarction Syndrome, Myocardial): ti,ab,kw 33

#5 (Preinfarction Syndromes, Myocardial): ti,ab,kw OR (Syndrome, Myocardial Preinfarction):ti,ab,kw OR (Syndromes, Myocardial Preinfarction):ti,ab,kw 7

#6 #1OR#2OR#3OR#4OR#5 5172

#7 (tongxinluo*): ti,ab,kw OR (Tongxinluo*):ti,ab,kw OR (TXL*):ti,ab,kw OR (Tong-xin-luo*):ti,ab,kw OR (Tong xin luo*):ti,ab,kw 190

#8 (Tong Xin Luo*): ti,ab,kw 22

#9 #7OR#8 190

#10 #6AND#9 31

**2.EMBASE Database**

( 'tongxinluo capsule': ab,ti OR 'tong xin luo*':ab,ti OR 'tong-xin-luo*':ab,ti OR 'txl*':ab,ti OR 'tongxinluo*':ab,ti ) AND ('random':ab,ti OR 'placebo':ab,ti OR 'double-blind':ab,ti )

31

**3.PubMed**

#1

Search: ("tongxinluo" [Supplementary Concept]) OR ((((((((tongxinluo*[Title/Abstract]) OR (tong xin luo*[Title/Abstract])) OR (TXL*[Title/Abstract])) OR (Tongxinluo*[Title/Abstract])) OR (Tong xin luo*[Title/Abstract])) OR (Tong Xin Luo*[Title/Abstract])) OR (Tong-Xin-Luo*[Title/Abstract])) OR (tong-xin-luo*[Title/Abstract]))

355

#2

(((Randomized Controlled Trial[ptyp])) OR ((Controlled Clinical Trial[ptyp])) OR ((Clinical Trial[ptyp])) OR ("Clinical Trials as Topic"[Mesh]) OR ("Clinical Trials, Phase III as Topic"[Mesh]) OR ("Clinical Trials, Phase IV as Topic"[Mesh]) OR ("Controlled Clinical Trials as Topic"[Mesh]) OR ("Clinical Trial"[Publication Type]) OR ("Controlled Clinical Trial"[Publication Type]) OR ("Clinical Trial, Phase III"[Publication Type]) OR ("Clinical Trial, Phase IV"[Publication Type]) OR ("Multicenter Study"[Publication Type]) OR ("Multicenter Studies as Topic"[Mesh]) OR ("Random Allocation"[Mesh]) OR ("Double-Blind Method"[Mesh]) OR ("Single-Blind Method"[Mesh]) OR ("Cross-Over Studies"[Mesh]) OR ("Placebos"[Mesh]) OR (controlled[tiab] AND (trial[tiab] OR trials[tiab] OR study[tiab] OR studies[tiab])) OR (blind[tiab] OR blinding[tiab] OR blinded[tiab] OR mask[tiab] OR masking[tiab] OR masked[tiab] OR placebo[tiab] OR placebos[tiab] OR rct[tiab] OR random[tiab] OR randomised[tiab] OR randomized[tiab] OR randomly[tiab] OR randomisation[tiab] OR randomization[tiab]) OR (factorial[tiab]) OR (divided[tiab] AND (group[tiab] OR groups[tiab])) OR (crossover[tiab]) OR ("cross over"[tiab]) OR (multicentre[tiab] OR multicentred[tiab] OR multicentric[tiab]) OR (versus[ti] OR vs[ti]) OR ("treatment arm"[tiab]) OR ("phase III"[tiab] OR "phase three"[tiab] OR "phase 3"[tiab]) OR ("latin square"[tiab]) NOT (("Animals"[Mesh] OR mouse[ti] OR mice[ti] OR pig[ti] OR pigs[ti] OR rat[ti] OR rats[ti] OR rabbit*[ti]) NOT (("Animals"[Mesh] OR mouse[ti] OR mice[ti] OR pig[ti] OR pigs[ti] OR rat[ti] OR rats[ti] OR rabbit*[ti] OR cadaver[ti] OR cadavers[ti]) AND ("Humans"[Mesh])))) [2,652,0](https://pubmed.ncbi.nlm.nih.gov/?term=(((Randomized+Controlled+Trial%5bptyp%5d))+OR+((Controlled+Clinical+Trial%5bptyp%5d))+OR+((Clinical+Trial%5bptyp%5d))+OR+()06

#3 #1AND#2 79

**4.SinoMed**

[(不稳定型心绞痛 OR 不稳定性心绞痛 OR 不稳定心绞痛 OR 不稳定型心绞病 OR 不稳定性心绞病 OR 不稳定心绞病 OR (初发*劳累*心绞痛) OR (初发劳力*心绞痛) OR (恶化*劳累*心绞痛) OR (恶化*心绞痛) OR (自发*心绞痛) OR (静息*心绞痛) OR (初发*心绞痛) OR (恶化*劳力*心绞痛) OR 急性冠脉综合征 OR 急性冠状动脉功能不全 OR 梗死性心绞痛 OR 梗死型心绞痛 OR 心肌梗塞前综合征 OR 心肌梗死前综合征 OR 心梗前综合征 OR 变异性心绞痛 OR 冠心病变异性心绞痛 OR 变异型心绞痛 OR 冠心病变异型心绞痛 OR 冠心病 OR 心绞痛 OR 冠状动脉粥样硬化性心脏病 OR 心肌梗死 OR 胸痹 OR 心痛 OR 静息心绞痛伴心电图缺血改变 OR 静息性心绞痛伴心电图缺血改变 OR 缺血改变 OR 心肌梗死后早期心绞痛 OR 心梗后早期心绞痛 OR 心肌梗塞后早期心绞痛) AND (通心络 OR 痛心络 OR 通欣络 OR 通新络 OR 通心洛) AND 随机](http://www.sinomed.ac.cn/javascript:toDoRelimitSearch();)

**5.CNKI**

（主题：不稳定型心绞痛 + 不稳定性心绞痛 + 不稳定心绞痛 + 不稳定型心绞病 + 不稳定性心绞病 + 不稳定心绞病）OR（主题："初发*劳累*心绞痛" + "初发劳力*心绞痛" + "恶化*劳累*心绞痛" + "恶化*心绞痛" + "自发*心绞痛" + "静息*心绞痛" + "初发*心绞痛" + "恶化*劳力*心绞痛"）OR（主题：急性冠脉综合征 + 急性冠状动脉功能不全 + 梗死性心绞痛 + 梗死型心绞痛 + 心肌梗塞前综合征 + 心肌梗死前综合征 + 心梗前综合征）OR（主题：变异性心绞痛 + 冠心病变异性心绞痛 + 变异型心绞痛 + 冠心病变异型心绞痛 + 冠心病 + 心绞痛 + 冠状动脉粥样硬化性心脏病 + 心肌梗死）OR（主题：胸痹 + 心痛）OR（主题：静息心绞痛伴心电图缺血改变 + 静息性心绞痛伴心电图缺血改变 + 缺血改变 + 心肌梗死后早期心绞痛 + 心梗后早期心绞痛 + 心肌梗塞后早期心绞痛） AND （（主题：通心络）OR（主题：痛心络）OR（主题：通欣络）OR（主题：通新络）OR（主题：通心洛）） AND （（全文：随机））

**6.Wanfang Database**

主题:(不稳定型心绞痛+不稳定性心绞痛+不稳定心绞痛+不稳定型心绞病+不稳定性心绞病+不稳定心绞病+“初发*劳累*心绞痛”+“初发劳力*心绞痛”+“恶化*劳累*心绞痛”+“恶化*心绞痛”+ “自发*心绞痛”+“静息*心绞痛”+“初发*心绞痛”+“恶化*劳力*心绞痛”+急性冠脉综合征+急性冠状动脉功能不全+梗死性心绞痛+梗死型心绞痛+心肌梗塞前综合征+心肌梗死前综合征+ 心梗前综合征+变异性心绞痛+冠心病变异性心绞痛+变异型心绞痛+冠心病变异型心绞痛+ 冠心病+心绞痛+冠状动脉粥样硬化性心脏病+心肌梗死+胸痹+心痛+静息心绞痛伴心电图缺血改变+静息性心绞痛伴心电图缺血改变+缺血改变+心肌梗死后早期心绞痛+心梗后早期心绞痛+心肌梗塞后早期心绞痛)*主题:(通心络+痛心络+通欣络+通新络+通心洛)*全部:(随机)

**7.VIP**

M=((不稳定型心绞痛 OR 不稳定性心绞痛 OR 不稳定心绞痛 OR 不稳定型心绞病 OR 不稳定性心绞病 OR 不稳定心绞病 OR (初发*劳累*心绞痛) OR (初发劳力*心绞痛) OR (恶化*劳累*心绞痛) OR (恶化*心绞痛) OR (自发*心绞痛) OR (静息*心绞痛) OR (初发*心绞痛) OR (恶化*劳力*心绞痛) OR 急性冠脉综合征 OR 急性冠状动脉功能不全 OR 梗死性心绞痛 OR 梗死型心绞痛 OR 心肌梗塞前综合征 OR 心肌梗死前综合征 OR 心梗前综合征 OR 变异性心绞痛 OR 冠心病变异性心绞痛 OR 变异型心绞痛 OR 冠心病变异型心绞痛 OR 冠心病 OR 心绞痛 OR 冠状动脉粥样硬化性心脏病 OR 心肌梗死 OR 胸痹 OR 心痛 OR 静息心绞痛伴心电图缺血改变 OR 静息性心绞痛伴心电图缺血改变 OR 缺血改变 OR 心肌梗死后早期心绞痛 OR 心梗后早期心绞痛 OR 心肌梗塞后早期心绞痛) AND (通心络 OR 痛心络 OR 通欣络 OR 通新络 OR 通心洛))
