# Supplementary material for: Efficacy and Safety of Tongxinluo Capsule as Adjunctive Treatment for Unstable Angina Pectoris: A Systematic Review and Meta-Analysis of Randomized Controlled Trials
Source: Front Pharmacol. 2021 Oct 11;12:742978. doi: 10.3389/fphar.2021.742978 (PMC8544810; doi:10.3389/fphar.2021.742978)
Supplement: Supplementary file 1 [file DataSheet1.zip › Supplementary material 3. The details of Tongxinluo capsule of all the included studies.docx]

***Supplementary material 1.***

The details of Tongxinluo capsule of all the included studies

| **Study** | **Formulation** | **Source** | **Species, concentration** | **Quality control reported**  **(Y/N)** | **Chemical analysis reported**  **(Y/N)** |
| --- | --- | --- | --- | --- | --- |
| Cai Zhelong 2010 | Tongxinluo Capsule: Ren Shen, Shui Zhi, Wu Gong, Tu Bie Chong, Quan Xie, Chan Tui, Chi Shao, Bing Pian, Tan Xiang, Ru Xiang, Jiang Xiang, Suan Zao Ren | Shijiazhuang Yiling Pharmaceutical Co., Ltd. | *Panax ginseng* C. A. Mey., *Hirudo nipponica* Whitman, *Scolopendra subspinipes mutilans* L. Koch, *Eupolyphaga sinensis* Walker, *Buthus martensii* Karsch, *Cryptotympana pustulata* Fabricius, *Paeonia lactiflora* Pall., *Dryobalanops aromatica* C.F.Gaertn., *Santalum album* L., *Boswellia carterii* Birdw., *Dalbergia odorifera* T.C.Chen, *Ziziphus jujuba* Mill. var. *spinosa*（Bunge）Hu ex H. F. Chou, concentration uncertainty of all ingredients | N | N |
| Chang Guodong 2018 | Tongxinluo Capsule: Ren Shen, Shui Zhi, Wu Gong, Tu Bie Chong, Quan Xie, Chan Tui, Chi Shao, Bing Pian, Tan Xiang, Ru Xiang, Jiang Xiang, Suan Zao Ren | Shijiazhuang Yiling Pharmaceutical Co., Ltd. | *Panax ginseng* C. A. Mey., *Hirudo nipponica* Whitman, *Scolopendra subspinipes mutilans* L. Koch, *Eupolyphaga sinensis* Walker, *Buthus martensii* Karsch, *Cryptotympana pustulata* Fabricius, *Paeonia lactiflora* Pall., *Dryobalanops aromatica* C.F.Gaertn., *Santalum album* L., *Boswellia carterii* Birdw., *Dalbergia odorifera* T.C.Chen, *Ziziphus jujuba* Mill. var. *spinosa*（Bunge）Hu ex H. F. Chou, concentration uncertainty of all ingredients | Y – Prepared in accordance with the standards of the National Medical Products Administration, approval number【Z19980015】 | N |
| Chang Shufang 2004 | Tongxinluo Capsule: Ren Shen, Shui Zhi, Wu Gong, Tu Bie Chong, Quan Xie, Chan Tui, Chi Shao, Bing Pian, Tan Xiang, Ru Xiang, Jiang Xiang, Suan Zao Ren | Shijiazhuang Yiling Pharmaceutical Co., Ltd. | *Panax ginseng* C. A. Mey., *Hirudo nipponica* Whitman, *Scolopendra subspinipes mutilans* L. Koch, *Eupolyphaga sinensis* Walker, *Buthus martensii* Karsch, *Cryptotympana pustulata* Fabricius, *Paeonia lactiflora* Pall., *Dryobalanops aromatica* C.F.Gaertn., *Santalum album* L., *Boswellia carterii* Birdw., *Dalbergia odorifera* T.C.Chen, *Ziziphus jujuba* Mill. var. *spinosa*（Bunge）Hu ex H. F. Chou, concentration uncertainty of all ingredients | N | N |
| Chen Rongxing2009 | Tongxinluo Capsule: Ren Shen, Shui Zhi, Wu Gong, Tu Bie Chong, Quan Xie, Chan Tui, Chi Shao, Bing Pian, Tan Xiang, Ru Xiang, Jiang Xiang, Suan Zao Ren | Shijiazhuang Yiling Pharmaceutical Co., Ltd. | *Panax ginseng* C. A. Mey., *Hirudo nipponica* Whitman, *Scolopendra subspinipes mutilans* L. Koch, *Eupolyphaga sinensis* Walker, *Buthus martensii* Karsch, *Cryptotympana pustulata* Fabricius, *Paeonia lactiflora* Pall., *Dryobalanops aromatica* C.F.Gaertn., *Santalum album* L., *Boswellia carterii* Birdw., *Dalbergia odorifera* T.C.Chen, *Ziziphus jujuba* Mill. var. *spinosa*（Bunge）Hu ex H. F. Chou, concentration uncertainty of all ingredients | N | N |
| Cui Dongmei 2008 | Tongxinluo Capsule: Ren Shen, Shui Zhi, Wu Gong, Tu Bie Chong, Quan Xie, Chan Tui, Chi Shao, Bing Pian, Tan Xiang, Ru Xiang, Jiang Xiang, Suan Zao Ren | Shijiazhuang Yiling Pharmaceutical Co., Ltd. | *Panax ginseng* C. A. Mey., *Hirudo nipponica* Whitman, *Scolopendra subspinipes mutilans* L. Koch, *Eupolyphaga sinensis* Walker, *Buthus martensii* Karsch, *Cryptotympana pustulata* Fabricius, *Paeonia lactiflora* Pall., *Dryobalanops aromatica* C.F.Gaertn., *Santalum album* L., *Boswellia carterii* Birdw., *Dalbergia odorifera* T.C.Chen, *Ziziphus jujuba* Mill. var. *spinosa*（Bunge）Hu ex H. F. Chou, concentration uncertainty of all ingredients | N | N |
| Ding Bo2013 | Tongxinluo Capsule: Ren Shen, Shui Zhi, Wu Gong, Tu Bie Chong, Quan Xie, Chan Tui, Chi Shao, Bing Pian, Tan Xiang, Ru Xiang, Jiang Xiang, Suan Zao Ren | Shijiazhuang Yiling Pharmaceutical Co., Ltd. | *Panax ginseng* C. A. Mey., *Hirudo nipponica* Whitman, *Scolopendra subspinipes mutilans* L. Koch, *Eupolyphaga sinensis* Walker, *Buthus martensii* Karsch, *Cryptotympana pustulata* Fabricius, *Paeonia lactiflora* Pall., *Dryobalanops aromatica* C.F.Gaertn., *Santalum album* L., *Boswellia carterii* Birdw., *Dalbergia odorifera* T.C.Chen, *Ziziphus jujuba* Mill. var. *spinosa*（Bunge）Hu ex H. F. Chou, concentration uncertainty of all ingredients | Y – Prepared in accordance with the standards of the National Medical Products Administration, approval number【Z19980015】 | N |
| Du Gaiyun2016 | Tongxinluo Capsule: Ren Shen, Shui Zhi, Wu Gong, Tu Bie Chong, Quan Xie, Chan Tui, Chi Shao, Bing Pian, Tan Xiang, Ru Xiang, Jiang Xiang, Suan Zao Ren | Shijiazhuang Yiling Pharmaceutical Co., Ltd. | *Panax ginseng* C. A. Mey., *Hirudo nipponica* Whitman, *Scolopendra subspinipes mutilans* L. Koch, *Eupolyphaga sinensis* Walker, *Buthus martensii* Karsch, *Cryptotympana pustulata* Fabricius, *Paeonia lactiflora* Pall., *Dryobalanops aromatica* C.F.Gaertn., *Santalum album* L., *Boswellia carterii* Birdw., *Dalbergia odorifera* T.C.Chen, *Ziziphus jujuba* Mill. var. *spinosa*（Bunge）Hu ex H. F. Chou, concentration uncertainty of all ingredients | N | N |
| Gao Jian2002 | Tongxinluo Capsule: Ren Shen, Shui Zhi, Wu Gong, Tu Bie Chong, Quan Xie, Chan Tui, Chi Shao, Bing Pian, Tan Xiang, Ru Xiang, Jiang Xiang, Suan Zao Ren | Shijiazhuang Yiling Pharmaceutical Co., Ltd. | *Panax ginseng* C. A. Mey., *Hirudo nipponica* Whitman, *Scolopendra subspinipes mutilans* L. Koch, *Eupolyphaga sinensis* Walker, *Buthus martensii* Karsch, *Cryptotympana pustulata* Fabricius, *Paeonia lactiflora* Pall., *Dryobalanops aromatica* C.F.Gaertn., *Santalum album* L., *Boswellia carterii* Birdw., *Dalbergia odorifera* T.C.Chen, *Ziziphus jujuba* Mill. var. *spinosa*（Bunge）Hu ex H. F. Chou, concentration uncertainty of all ingredients | N | N |
| Hao Rongjing2015 | Tongxinluo Capsule: Ren Shen, Shui Zhi, Wu Gong, Tu Bie Chong, Quan Xie, Chan Tui, Chi Shao, Bing Pian, Tan Xiang, Ru Xiang, Jiang Xiang, Suan Zao Ren | Shijiazhuang Yiling Pharmaceutical Co., Ltd. | *Panax ginseng* C. A. Mey., *Hirudo nipponica* Whitman, *Scolopendra subspinipes mutilans* L. Koch, *Eupolyphaga sinensis* Walker, *Buthus martensii* Karsch, *Cryptotympana pustulata* Fabricius, *Paeonia lactiflora* Pall., *Dryobalanops aromatica* C.F.Gaertn., *Santalum album* L., *Boswellia carterii* Birdw., *Dalbergia odorifera* T.C.Chen, *Ziziphus jujuba* Mill. var. *spinosa*（Bunge）Hu ex H. F. Chou, concentration uncertainty of all ingredients | N | N |
| Hui hui 2018 | Tongxinluo Capsule: Ren Shen, Shui Zhi, Wu Gong, Tu Bie Chong, Quan Xie, Chan Tui, Chi Shao, Bing Pian, Tan Xiang, Ru Xiang, Jiang Xiang, Suan Zao Ren | Shijiazhuang Yiling Pharmaceutical Co., Ltd. | *Panax ginseng* C. A. Mey., *Hirudo nipponica* Whitman, *Scolopendra subspinipes mutilans* L. Koch, *Eupolyphaga sinensis* Walker, *Buthus martensii* Karsch, *Cryptotympana pustulata* Fabricius, *Paeonia lactiflora* Pall., *Dryobalanops aromatica* C.F.Gaertn., *Santalum album* L., *Boswellia carterii* Birdw., *Dalbergia odorifera* T.C.Chen, *Ziziphus jujuba* Mill. var. *spinosa*（Bunge）Hu ex H. F. Chou, concentration uncertainty of all ingredients | Y – Prepared in accordance with the standards of the National Medical Products Administration, approval number【Z19980015】 | N |
| Jiang Haiyan 2019 | Tongxinluo Capsule: Ren Shen, Shui Zhi, Wu Gong, Tu Bie Chong, Quan Xie, Chan Tui, Chi Shao, Bing Pian, Tan Xiang, Ru Xiang, Jiang Xiang, Suan Zao Ren | Shijiazhuang Yiling Pharmaceutical Co., Ltd. | *Panax ginseng* C. A. Mey., *Hirudo nipponica* Whitman, *Scolopendra subspinipes mutilans* L. Koch, *Eupolyphaga sinensis* Walker, *Buthus martensii* Karsch, *Cryptotympana pustulata* Fabricius, *Paeonia lactiflora* Pall., *Dryobalanops aromatica* C.F.Gaertn., *Santalum album* L., *Boswellia carterii* Birdw., *Dalbergia odorifera* T.C.Chen, *Ziziphus jujuba* Mill. var. *spinosa*（Bunge）Hu ex H. F. Chou, concentration uncertainty of all ingredients | N | N |
| Li Quan2018 | Tongxinluo Capsule: Ren Shen, Shui Zhi, Wu Gong, Tu Bie Chong, Quan Xie, Chan Tui, Chi Shao, Bing Pian, Tan Xiang, Ru Xiang, Jiang Xiang, Suan Zao Ren | Shijiazhuang Yiling Pharmaceutical Co., Ltd. | *Panax ginseng* C. A. Mey., *Hirudo nipponica* Whitman, *Scolopendra subspinipes mutilans* L. Koch, *Eupolyphaga sinensis* Walker, *Buthus martensii* Karsch, *Cryptotympana pustulata* Fabricius, *Paeonia lactiflora* Pall., *Dryobalanops aromatica* C.F.Gaertn., *Santalum album* L., *Boswellia carterii* Birdw., *Dalbergia odorifera* T.C.Chen, *Ziziphus jujuba* Mill. var. *spinosa*（Bunge）Hu ex H. F. Chou, concentration uncertainty of all ingredients | N | N |
| Li Ting 2021 | Tongxinluo Capsule: Ren Shen, Shui Zhi, Wu Gong, Tu Bie Chong, Quan Xie, Chan Tui, Chi Shao, Bing Pian, Tan Xiang, Ru Xiang, Jiang Xiang, Suan Zao Ren | Shijiazhuang Yiling Pharmaceutical Co., Ltd. | *Panax ginseng* C. A. Mey., *Hirudo nipponica* Whitman, *Scolopendra subspinipes mutilans* L. Koch, *Eupolyphaga sinensis* Walker, *Buthus martensii* Karsch, *Cryptotympana pustulata* Fabricius, *Paeonia lactiflora* Pall., *Dryobalanops aromatica* C.F.Gaertn., *Santalum album* L., *Boswellia carterii* Birdw., *Dalbergia odorifera* T.C.Chen, *Ziziphus jujuba* Mill. var. *spinosa*（Bunge）Hu ex H. F. Chou, concentration uncertainty of all ingredients | N | N |
| Li Xiaocheng 2013 | Tongxinluo Capsule: Ren Shen, Shui Zhi, Wu Gong, Tu Bie Chong, Quan Xie, Chan Tui, Chi Shao, Bing Pian, Tan Xiang, Ru Xiang, Jiang Xiang, Suan Zao Ren | Shijiazhuang Yiling Pharmaceutical Co., Ltd. | *Panax ginseng* C. A. Mey., *Hirudo nipponica* Whitman, *Scolopendra subspinipes mutilans* L. Koch, *Eupolyphaga sinensis* Walker, *Buthus martensii* Karsch, *Cryptotympana pustulata* Fabricius, *Paeonia lactiflora* Pall., *Dryobalanops aromatica* C.F.Gaertn., *Santalum album* L., *Boswellia carterii* Birdw., *Dalbergia odorifera* T.C.Chen, *Ziziphus jujuba* Mill. var. *spinosa*（Bunge）Hu ex H. F. Chou, concentration uncertainty of all ingredients | N | N |
| Liu Shuguang 2016 | Tongxinluo Capsule: Ren Shen, Shui Zhi, Wu Gong, Tu Bie Chong, Quan Xie, Chan Tui, Chi Shao, Bing Pian, Tan Xiang, Ru Xiang, Jiang Xiang, Suan Zao Ren | Shijiazhuang Yiling Pharmaceutical Co., Ltd. | *Panax ginseng* C. A. Mey., *Hirudo nipponica* Whitman, *Scolopendra subspinipes mutilans* L. Koch, *Eupolyphaga sinensis* Walker, *Buthus martensii* Karsch, *Cryptotympana pustulata* Fabricius, *Paeonia lactiflora* Pall., *Dryobalanops aromatica* C.F.Gaertn., *Santalum album* L., *Boswellia carterii* Birdw., *Dalbergia odorifera* T.C.Chen, *Ziziphus jujuba* Mill. var. *spinosa*（Bunge）Hu ex H. F. Chou, concentration uncertainty of all ingredients | Y – Prepared in accordance with the standards of the National Medical Products Administration, approval number【Z19980015】 | N |
| Liu Yuanxin 2011 | Tongxinluo Capsule: Ren Shen, Shui Zhi, Wu Gong, Tu Bie Chong, Quan Xie, Chan Tui, Chi Shao, Bing Pian, Tan Xiang, Ru Xiang, Jiang Xiang, Suan Zao Ren | Shijiazhuang Yiling Pharmaceutical Co., Ltd. | *Panax ginseng* C. A. Mey., *Hirudo nipponica* Whitman, *Scolopendra subspinipes mutilans* L. Koch, *Eupolyphaga sinensis* Walker, *Buthus martensii* Karsch, *Cryptotympana pustulata* Fabricius, *Paeonia lactiflora* Pall., *Dryobalanops aromatica* C.F.Gaertn., *Santalum album* L., *Boswellia carterii* Birdw., *Dalbergia odorifera* T.C.Chen, *Ziziphus jujuba* Mill. var. *spinosa*（Bunge）Hu ex H. F. Chou, concentration uncertainty of all ingredients | N | N |
| Luo Han 2013 | Tongxinluo Capsule: Ren Shen, Shui Zhi, Wu Gong, Tu Bie Chong, Quan Xie, Chan Tui, Chi Shao, Bing Pian, Tan Xiang, Ru Xiang, Jiang Xiang, Suan Zao Ren | Shijiazhuang Yiling Pharmaceutical Co., Ltd. | *Panax ginseng* C. A. Mey., *Hirudo nipponica* Whitman, *Scolopendra subspinipes mutilans* L. Koch, *Eupolyphaga sinensis* Walker, *Buthus martensii* Karsch, *Cryptotympana pustulata* Fabricius, *Paeonia lactiflora* Pall., *Dryobalanops aromatica* C.F.Gaertn., *Santalum album* L., *Boswellia carterii* Birdw., *Dalbergia odorifera* T.C.Chen, *Ziziphus jujuba* Mill. var. *spinosa*（Bunge）Hu ex H. F. Chou, concentration uncertainty of all ingredients | Y – Prepared in accordance with the standards of the National Medical Products Administration, approval number【Z19980015】 | N |
| Ma jinying 2011 | Tongxinluo Capsule: Ren Shen, Shui Zhi, Wu Gong, Tu Bie Chong, Quan Xie, Chan Tui, Chi Shao, Bing Pian, Tan Xiang, Ru Xiang, Jiang Xiang, Suan Zao Ren | Shijiazhuang Yiling Pharmaceutical Co., Ltd. | *Panax ginseng* C. A. Mey., *Hirudo nipponica* Whitman, *Scolopendra subspinipes mutilans* L. Koch, *Eupolyphaga sinensis* Walker, *Buthus martensii* Karsch, *Cryptotympana pustulata* Fabricius, *Paeonia lactiflora* Pall., *Dryobalanops aromatica* C.F.Gaertn., *Santalum album* L., *Boswellia carterii* Birdw., *Dalbergia odorifera* T.C.Chen, *Ziziphus jujuba* Mill. var. *spinosa*（Bunge）Hu ex H. F. Chou, concentration uncertainty of all ingredients | N | N |
| Ren jingjuan 2018 | Tongxinluo Capsule: Ren Shen, Shui Zhi, Wu Gong, Tu Bie Chong, Quan Xie, Chan Tui, Chi Shao, Bing Pian, Tan Xiang, Ru Xiang, Jiang Xiang, Suan Zao Ren | Shijiazhuang Yiling Pharmaceutical Co., Ltd. | *Panax ginseng* C. A. Mey., *Hirudo nipponica* Whitman, *Scolopendra subspinipes mutilans* L. Koch, *Eupolyphaga sinensis* Walker, *Buthus martensii* Karsch, *Cryptotympana pustulata* Fabricius, *Paeonia lactiflora* Pall., *Dryobalanops aromatica* C.F.Gaertn., *Santalum album* L., *Boswellia carterii* Birdw., *Dalbergia odorifera* T.C.Chen, *Ziziphus jujuba* Mill. var. *spinosa*（Bunge）Hu ex H. F. Chou, concentration uncertainty of all ingredients | Y – Prepared in accordance with the standards of the National Medical Products Administration, approval number【Z19980015】 | N |
| Shi Chunqing 2013 | Tongxinluo Capsule: Ren Shen, Shui Zhi, Wu Gong, Tu Bie Chong, Quan Xie, Chan Tui, Chi Shao, Bing Pian, Tan Xiang, Ru Xiang, Jiang Xiang, Suan Zao Ren | Shijiazhuang Yiling Pharmaceutical Co., Ltd. | *Panax ginseng* C. A. Mey., *Hirudo nipponica* Whitman, *Scolopendra subspinipes mutilans* L. Koch, *Eupolyphaga sinensis* Walker, *Buthus martensii* Karsch, *Cryptotympana pustulata* Fabricius, *Paeonia lactiflora* Pall., *Dryobalanops aromatica* C.F.Gaertn., *Santalum album* L., *Boswellia carterii* Birdw., *Dalbergia odorifera* T.C.Chen, *Ziziphus jujuba* Mill. var. *spinosa*（Bunge）Hu ex H. F. Chou, concentration uncertainty of all ingredients | N | N |
| Song Kai 2008 | Tongxinluo Capsule: Ren Shen, Shui Zhi, Wu Gong, Tu Bie Chong, Quan Xie, Chan Tui, Chi Shao, Bing Pian, Tan Xiang, Ru Xiang, Jiang Xiang, Suan Zao Ren | Shijiazhuang Yiling Pharmaceutical Co., Ltd. | *Panax ginseng* C. A. Mey., *Hirudo nipponica* Whitman, *Scolopendra subspinipes mutilans* L. Koch, *Eupolyphaga sinensis* Walker, *Buthus martensii* Karsch, *Cryptotympana pustulata* Fabricius, *Paeonia lactiflora* Pall., *Dryobalanops aromatica* C.F.Gaertn., *Santalum album* L., *Boswellia carterii* Birdw., *Dalbergia odorifera* T.C.Chen, *Ziziphus jujuba* Mill. var. *spinosa*（Bunge）Hu ex H. F. Chou, concentration uncertainty of all ingredients | N | N |
| Sun Guangjiang 2011 | Tongxinluo Capsule: Ren Shen, Shui Zhi, Wu Gong, Tu Bie Chong, Quan Xie, Chan Tui, Chi Shao, Bing Pian, Tan Xiang, Ru Xiang, Jiang Xiang, Suan Zao Ren | Shijiazhuang Yiling Pharmaceutical Co., Ltd. | *Panax ginseng* C. A. Mey., *Hirudo nipponica* Whitman, *Scolopendra subspinipes mutilans* L. Koch, *Eupolyphaga sinensis* Walker, *Buthus martensii* Karsch, *Cryptotympana pustulata* Fabricius, *Paeonia lactiflora* Pall., *Dryobalanops aromatica* C.F.Gaertn., *Santalum album* L., *Boswellia carterii* Birdw., *Dalbergia odorifera* T.C.Chen, *Ziziphus jujuba* Mill. var. *spinosa*（Bunge）Hu ex H. F. Chou, concentration uncertainty of all ingredients | N | N |
| Tian Chuanxin 2005 | Tongxinluo Capsule: Ren Shen, Shui Zhi, Wu Gong, Tu Bie Chong, Quan Xie, Chan Tui, Chi Shao, Bing Pian, Tan Xiang, Ru Xiang, Jiang Xiang, Suan Zao Ren | Shijiazhuang Yiling Pharmaceutical Co., Ltd. | *Panax ginseng* C. A. Mey., *Hirudo nipponica* Whitman, *Scolopendra subspinipes mutilans* L. Koch, *Eupolyphaga sinensis* Walker, *Buthus martensii* Karsch, *Cryptotympana pustulata* Fabricius, *Paeonia lactiflora* Pall., *Dryobalanops aromatica* C.F.Gaertn., *Santalum album* L., *Boswellia carterii* Birdw., *Dalbergia odorifera* T.C.Chen, *Ziziphus jujuba* Mill. var. *spinosa*（Bunge）Hu ex H. F. Chou, concentration uncertainty of all ingredients | N | N |
| Tian Fengxuan 2007 | Tongxinluo Capsule: Ren Shen, Shui Zhi, Wu Gong, Tu Bie Chong, Quan Xie, Chan Tui, Chi Shao, Bing Pian, Tan Xiang, Ru Xiang, Jiang Xiang, Suan Zao Ren | Shijiazhuang Yiling Pharmaceutical Co., Ltd. | *Panax ginseng* C. A. Mey., *Hirudo nipponica* Whitman, *Scolopendra subspinipes mutilans* L. Koch, *Eupolyphaga sinensis* Walker, *Buthus martensii* Karsch, *Cryptotympana pustulata* Fabricius, *Paeonia lactiflora* Pall., *Dryobalanops aromatica* C.F.Gaertn., *Santalum album* L., *Boswellia carterii* Birdw., *Dalbergia odorifera* T.C.Chen, *Ziziphus jujuba* Mill. var. *spinosa*（Bunge）Hu ex H. F. Chou, concentration uncertainty of all ingredients | N | N |
| Wang Caiping 2007 | Tongxinluo Capsule: Ren Shen, Shui Zhi, Wu Gong, Tu Bie Chong, Quan Xie, Chan Tui, Chi Shao, Bing Pian, Tan Xiang, Ru Xiang, Jiang Xiang, Suan Zao Ren | Shijiazhuang Yiling Pharmaceutical Co., Ltd. | *Panax ginseng* C. A. Mey., *Hirudo nipponica* Whitman, *Scolopendra subspinipes mutilans* L. Koch, *Eupolyphaga sinensis* Walker, *Buthus martensii* Karsch, *Cryptotympana pustulata* Fabricius, *Paeonia lactiflora* Pall., *Dryobalanops aromatica* C.F.Gaertn., *Santalum album* L., *Boswellia carterii* Birdw., *Dalbergia odorifera* T.C.Chen, *Ziziphus jujuba* Mill. var. *spinosa*（Bunge）Hu ex H. F. Chou, concentration uncertainty of all ingredients | N | N |
| Wang Huan 2017 | Tongxinluo Capsule: Ren Shen, Shui Zhi, Wu Gong, Tu Bie Chong, Quan Xie, Chan Tui, Chi Shao, Bing Pian, Tan Xiang, Ru Xiang, Jiang Xiang, Suan Zao Ren | Shijiazhuang Yiling Pharmaceutical Co., Ltd. | *Panax ginseng* C. A. Mey., *Hirudo nipponica* Whitman, *Scolopendra subspinipes mutilans* L. Koch, *Eupolyphaga sinensis* Walker, *Buthus martensii* Karsch, *Cryptotympana pustulata* Fabricius, *Paeonia lactiflora* Pall., *Dryobalanops aromatica* C.F.Gaertn., *Santalum album* L., *Boswellia carterii* Birdw., *Dalbergia odorifera* T.C.Chen, *Ziziphus jujuba* Mill. var. *spinosa*（Bunge）Hu ex H. F. Chou, concentration uncertainty of all ingredients | N | N |
| Wang Lixin 2013 | Tongxinluo Capsule: Ren Shen, Shui Zhi, Wu Gong, Tu Bie Chong, Quan Xie, Chan Tui, Chi Shao, Bing Pian, Tan Xiang, Ru Xiang, Jiang Xiang, Suan Zao Ren | Shijiazhuang Yiling Pharmaceutical Co., Ltd. | *Panax ginseng* C. A. Mey., *Hirudo nipponica* Whitman, *Scolopendra subspinipes mutilans* L. Koch, *Eupolyphaga sinensis* Walker, *Buthus martensii* Karsch, *Cryptotympana pustulata* Fabricius, *Paeonia lactiflora* Pall., *Dryobalanops aromatica* C.F.Gaertn., *Santalum album* L., *Boswellia carterii* Birdw., *Dalbergia odorifera* T.C.Chen, *Ziziphus jujuba* Mill. var. *spinosa*（Bunge）Hu ex H. F. Chou, concentration uncertainty of all ingredients | N | N |
| Wang Shixun 2010 | Tongxinluo Capsule: Ren Shen, Shui Zhi, Wu Gong, Tu Bie Chong, Quan Xie, Chan Tui, Chi Shao, Bing Pian, Tan Xiang, Ru Xiang, Jiang Xiang, Suan Zao Ren | Shijiazhuang Yiling Pharmaceutical Co., Ltd. | *Panax ginseng* C. A. Mey., *Hirudo nipponica* Whitman, *Scolopendra subspinipes mutilans* L. Koch, *Eupolyphaga sinensis* Walker, *Buthus martensii* Karsch, *Cryptotympana pustulata* Fabricius, *Paeonia lactiflora* Pall., *Dryobalanops aromatica* C.F.Gaertn., *Santalum album* L., *Boswellia carterii* Birdw., *Dalbergia odorifera* T.C.Chen, *Ziziphus jujuba* Mill. var. *spinosa*（Bunge）Hu ex H. F. Chou, concentration uncertainty of all ingredients | N | N |
| Wang Sujuan 2015 | Tongxinluo Capsule: Ren Shen, Shui Zhi, Wu Gong, Tu Bie Chong, Quan Xie, Chan Tui, Chi Shao, Bing Pian, Tan Xiang, Ru Xiang, Jiang Xiang, Suan Zao Ren | Shijiazhuang Yiling Pharmaceutical Co., Ltd. | *Panax ginseng* C. A. Mey., *Hirudo nipponica* Whitman, *Scolopendra subspinipes mutilans* L. Koch, *Eupolyphaga sinensis* Walker, *Buthus martensii* Karsch, *Cryptotympana pustulata* Fabricius, *Paeonia lactiflora* Pall., *Dryobalanops aromatica* C.F.Gaertn., *Santalum album* L., *Boswellia carterii* Birdw., *Dalbergia odorifera* T.C.Chen, *Ziziphus jujuba* Mill. var. *spinosa*（Bunge）Hu ex H. F. Chou, concentration uncertainty of all ingredients | N | N |
| Wang Xiaoping 2009 | Tongxinluo Capsule: Ren Shen, Shui Zhi, Wu Gong, Tu Bie Chong, Quan Xie, Chan Tui, Chi Shao, Bing Pian, Tan Xiang, Ru Xiang, Jiang Xiang, Suan Zao Ren | Shijiazhuang Yiling Pharmaceutical Co., Ltd. | *Panax ginseng* C. A. Mey., *Hirudo nipponica* Whitman, *Scolopendra subspinipes mutilans* L. Koch, *Eupolyphaga sinensis* Walker, *Buthus martensii* Karsch, *Cryptotympana pustulata* Fabricius, *Paeonia lactiflora* Pall., *Dryobalanops aromatica* C.F.Gaertn., *Santalum album* L., *Boswellia carterii* Birdw., *Dalbergia odorifera* T.C.Chen, *Ziziphus jujuba* Mill. var. *spinosa*（Bunge）Hu ex H. F. Chou, concentration uncertainty of all ingredients | N | N |
| Wang Zhenguo 2012 | Tongxinluo Capsule: Ren Shen, Shui Zhi, Wu Gong, Tu Bie Chong, Quan Xie, Chan Tui, Chi Shao, Bing Pian, Tan Xiang, Ru Xiang, Jiang Xiang, Suan Zao Ren | Shijiazhuang Yiling Pharmaceutical Co., Ltd. | *Panax ginseng* C. A. Mey., *Hirudo nipponica* Whitman, *Scolopendra subspinipes mutilans* L. Koch, *Eupolyphaga sinensis* Walker, *Buthus martensii* Karsch, *Cryptotympana pustulata* Fabricius, *Paeonia lactiflora* Pall., *Dryobalanops aromatica* C.F.Gaertn., *Santalum album* L., *Boswellia carterii* Birdw., *Dalbergia odorifera* T.C.Chen, *Ziziphus jujuba* Mill. var. *spinosa*（Bunge）Hu ex H. F. Chou, concentration uncertainty of all ingredients | N | N |
| Wu Chun 2011 | Tongxinluo Capsule: Ren Shen, Shui Zhi, Wu Gong, Tu Bie Chong, Quan Xie, Chan Tui, Chi Shao, Bing Pian, Tan Xiang, Ru Xiang, Jiang Xiang, Suan Zao Ren | Shijiazhuang Yiling Pharmaceutical Co., Ltd. | *Panax ginseng* C. A. Mey., *Hirudo nipponica* Whitman, *Scolopendra subspinipes mutilans* L. Koch, *Eupolyphaga sinensis* Walker, *Buthus martensii* Karsch, *Cryptotympana pustulata* Fabricius, *Paeonia lactiflora* Pall., *Dryobalanops aromatica* C.F.Gaertn., *Santalum album* L., *Boswellia carterii* Birdw., *Dalbergia odorifera* T.C.Chen, *Ziziphus jujuba* Mill. var. *spinosa*（Bunge）Hu ex H. F. Chou, concentration uncertainty of all ingredients | Y – Prepared in accordance with the standards of the National Medical Products Administration, approval number【Z19980015】 | N |
| Wu Songjiao 2006 | Tongxinluo Capsule: Ren Shen, Shui Zhi, Wu Gong, Tu Bie Chong, Quan Xie, Chan Tui, Chi Shao, Bing Pian, Tan Xiang, Ru Xiang, Jiang Xiang, Suan Zao Ren | Shijiazhuang Yiling Pharmaceutical Co., Ltd. | *Panax ginseng* C. A. Mey., *Hirudo nipponica* Whitman, *Scolopendra subspinipes mutilans* L. Koch, *Eupolyphaga sinensis* Walker, *Buthus martensii* Karsch, *Cryptotympana pustulata* Fabricius, *Paeonia lactiflora* Pall., *Dryobalanops aromatica* C.F.Gaertn., *Santalum album* L., *Boswellia carterii* Birdw., *Dalbergia odorifera* T.C.Chen, *Ziziphus jujuba* Mill. var. *spinosa*（Bunge）Hu ex H. F. Chou, concentration uncertainty of all ingredients | N | N |
| Wu Zongyi 2010 | Tongxinluo Capsule: Ren Shen, Shui Zhi, Wu Gong, Tu Bie Chong, Quan Xie, Chan Tui, Chi Shao, Bing Pian, Tan Xiang, Ru Xiang, Jiang Xiang, Suan Zao Ren | Shijiazhuang Yiling Pharmaceutical Co., Ltd. | *Panax ginseng* C. A. Mey., *Hirudo nipponica* Whitman, *Scolopendra subspinipes mutilans* L. Koch, *Eupolyphaga sinensis* Walker, *Buthus martensii* Karsch, *Cryptotympana pustulata* Fabricius, *Paeonia lactiflora* Pall., *Dryobalanops aromatica* C.F.Gaertn., *Santalum album* L., *Boswellia carterii* Birdw., *Dalbergia odorifera* T.C.Chen, *Ziziphus jujuba* Mill. var. *spinosa*（Bunge）Hu ex H. F. Chou, concentration uncertainty of all ingredients | N | N |
| Xin Ling 2008 | Tongxinluo Capsule: Ren Shen, Shui Zhi, Wu Gong, Tu Bie Chong, Quan Xie, Chan Tui, Chi Shao, Bing Pian, Tan Xiang, Ru Xiang, Jiang Xiang, Suan Zao Ren | Shijiazhuang Yiling Pharmaceutical Co., Ltd. | *Panax ginseng* C. A. Mey., *Hirudo nipponica* Whitman, *Scolopendra subspinipes mutilans* L. Koch, *Eupolyphaga sinensis* Walker, *Buthus martensii* Karsch, *Cryptotympana pustulata* Fabricius, *Paeonia lactiflora* Pall., *Dryobalanops aromatica* C.F.Gaertn., *Santalum album* L., *Boswellia carterii* Birdw., *Dalbergia odorifera* T.C.Chen, *Ziziphus jujuba* Mill. var. *spinosa*（Bunge）Hu ex H. F. Chou, concentration uncertainty of all ingredients | N | N |
| Xing Xuexin 2013 | Tongxinluo Capsule: Ren Shen, Shui Zhi, Wu Gong, Tu Bie Chong, Quan Xie, Chan Tui, Chi Shao, Bing Pian, Tan Xiang, Ru Xiang, Jiang Xiang, Suan Zao Ren | Shijiazhuang Yiling Pharmaceutical Co., Ltd. | *Panax ginseng* C. A. Mey., *Hirudo nipponica* Whitman, *Scolopendra subspinipes mutilans* L. Koch, *Eupolyphaga sinensis* Walker, *Buthus martensii* Karsch, *Cryptotympana pustulata* Fabricius, *Paeonia lactiflora* Pall., *Dryobalanops aromatica* C.F.Gaertn., *Santalum album* L., *Boswellia carterii* Birdw., *Dalbergia odorifera* T.C.Chen, *Ziziphus jujuba* Mill. var. *spinosa*（Bunge）Hu ex H. F. Chou, concentration uncertainty of all ingredients | N | N |
| Yang Fan 2008 | Tongxinluo Capsule: Ren Shen, Shui Zhi, Wu Gong, Tu Bie Chong, Quan Xie, Chan Tui, Chi Shao, Bing Pian, Tan Xiang, Ru Xiang, Jiang Xiang, Suan Zao Ren | Shijiazhuang Yiling Pharmaceutical Co., Ltd. | *Panax ginseng* C. A. Mey., *Hirudo nipponica* Whitman, *Scolopendra subspinipes mutilans* L. Koch, *Eupolyphaga sinensis* Walker, *Buthus martensii* Karsch, *Cryptotympana pustulata* Fabricius, *Paeonia lactiflora* Pall., *Dryobalanops aromatica* C.F.Gaertn., *Santalum album* L., *Boswellia carterii* Birdw., *Dalbergia odorifera* T.C.Chen, *Ziziphus jujuba* Mill. var. *spinosa*（Bunge）Hu ex H. F. Chou, concentration uncertainty of all ingredients | N | N |
| Yang Jidong 2019 | Tongxinluo Capsule: Ren Shen, Shui Zhi, Wu Gong, Tu Bie Chong, Quan Xie, Chan Tui, Chi Shao, Bing Pian, Tan Xiang, Ru Xiang, Jiang Xiang, Suan Zao Ren | Shijiazhuang Yiling Pharmaceutical Co., Ltd. | *Panax ginseng* C. A. Mey., *Hirudo nipponica* Whitman, *Scolopendra subspinipes mutilans* L. Koch, *Eupolyphaga sinensis* Walker, *Buthus martensii* Karsch, *Cryptotympana pustulata* Fabricius, *Paeonia lactiflora* Pall., *Dryobalanops aromatica* C.F.Gaertn., *Santalum album* L., *Boswellia carterii* Birdw., *Dalbergia odorifera* T.C.Chen, *Ziziphus jujuba* Mill. var. *spinosa*（Bunge）Hu ex H. F. Chou, concentration uncertainty of all ingredients | Y – Prepared in accordance with the standards of the National Medical Products Administration, approval number【Z19980015】 | N |
| Yu Meiling 2015 | Tongxinluo Capsule: Ren Shen, Shui Zhi, Wu Gong, Tu Bie Chong, Quan Xie, Chan Tui, Chi Shao, Bing Pian, Tan Xiang, Ru Xiang, Jiang Xiang, Suan Zao Ren | Shijiazhuang Yiling Pharmaceutical Co., Ltd. | *Panax ginseng* C. A. Mey., *Hirudo nipponica* Whitman, *Scolopendra subspinipes mutilans* L. Koch, *Eupolyphaga sinensis* Walker, *Buthus martensii* Karsch, *Cryptotympana pustulata* Fabricius, *Paeonia lactiflora* Pall., *Dryobalanops aromatica* C.F.Gaertn., *Santalum album* L., *Boswellia carterii* Birdw., *Dalbergia odorifera* T.C.Chen, *Ziziphus jujuba* Mill. var. *spinosa*（Bunge）Hu ex H. F. Chou, concentration uncertainty of all ingredients | N | N |
| Yu Yingsun 2012 | Tongxinluo Capsule: Ren Shen, Shui Zhi, Wu Gong, Tu Bie Chong, Quan Xie, Chan Tui, Chi Shao, Bing Pian, Tan Xiang, Ru Xiang, Jiang Xiang, Suan Zao Ren | Shijiazhuang Yiling Pharmaceutical Co., Ltd. | *Panax ginseng* C. A. Mey., *Hirudo nipponica* Whitman, *Scolopendra subspinipes mutilans* L. Koch, *Eupolyphaga sinensis* Walker, *Buthus martensii* Karsch, *Cryptotympana pustulata* Fabricius, *Paeonia lactiflora* Pall., *Dryobalanops aromatica* C.F.Gaertn., *Santalum album* L., *Boswellia carterii* Birdw., *Dalbergia odorifera* T.C.Chen, *Ziziphus jujuba* Mill. var. *spinosa*（Bunge）Hu ex H. F. Chou, concentration uncertainty of all ingredients | Y – Prepared in accordance with the standards of the National Medical Products Administration, approval number【Z19980015】 | N |
| Yuan Wenjie 2019 | Tongxinluo Capsule: Ren Shen, Shui Zhi, Wu Gong, Tu Bie Chong, Quan Xie, Chan Tui, Chi Shao, Bing Pian, Tan Xiang, Ru Xiang, Jiang Xiang, Suan Zao Ren | Shijiazhuang Yiling Pharmaceutical Co., Ltd. | *Panax ginseng* C. A. Mey., *Hirudo nipponica* Whitman, *Scolopendra subspinipes mutilans* L. Koch, *Eupolyphaga sinensis* Walker, *Buthus martensii* Karsch, *Cryptotympana pustulata* Fabricius, *Paeonia lactiflora* Pall., *Dryobalanops aromatica* C.F.Gaertn., *Santalum album* L., *Boswellia carterii* Birdw., *Dalbergia odorifera* T.C.Chen, *Ziziphus jujuba* Mill. var. *spinosa*（Bunge）Hu ex H. F. Chou, concentration uncertainty of all ingredients | Y – Prepared in accordance with the standards of the National Medical Products Administration, approval number【Z19980015】 | N |
| Zhang Jing2009 | Tongxinluo Capsule: Ren Shen, Shui Zhi, Wu Gong, Tu Bie Chong, Quan Xie, Chan Tui, Chi Shao, Bing Pian, Tan Xiang, Ru Xiang, Jiang Xiang, Suan Zao Ren | Shijiazhuang Yiling Pharmaceutical Co., Ltd. | *Panax ginseng* C. A. Mey., *Hirudo nipponica* Whitman, *Scolopendra subspinipes mutilans* L. Koch, *Eupolyphaga sinensis* Walker, *Buthus martensii* Karsch, *Cryptotympana pustulata* Fabricius, *Paeonia lactiflora* Pall., *Dryobalanops aromatica* C.F.Gaertn., *Santalum album* L., *Boswellia carterii* Birdw., *Dalbergia odorifera* T.C.Chen, *Ziziphus jujuba* Mill. var. *spinosa*（Bunge）Hu ex H. F. Chou, concentration uncertainty of all ingredients | N | N |
| Zhou Yumei 2013 | Tongxinluo Capsule: Ren Shen, Shui Zhi, Wu Gong, Tu Bie Chong, Quan Xie, Chan Tui, Chi Shao, Bing Pian, Tan Xiang, Ru Xiang, Jiang Xiang, Suan Zao Ren | Shijiazhuang Yiling Pharmaceutical Co., Ltd. | *Panax ginseng* C. A. Mey., *Hirudo nipponica* Whitman, *Scolopendra subspinipes mutilans* L. Koch, *Eupolyphaga sinensis* Walker, *Buthus martensii* Karsch, *Cryptotympana pustulata* Fabricius, *Paeonia lactiflora* Pall., *Dryobalanops aromatica* C.F.Gaertn., *Santalum album* L., *Boswellia carterii* Birdw., *Dalbergia odorifera* T.C.Chen, *Ziziphus jujuba* Mill. var. *spinosa*（Bunge）Hu ex H. F. Chou, concentration uncertainty of all ingredients | N | N |
