# Supplementary figures and images for: Efficacy and Safety of Tongxinluo Capsule as Adjunctive Treatment for Unstable Angina Pectoris: A Systematic Review and Meta-Analysis of Randomized Controlled Trials
Source: Front Pharmacol. 2021 Oct 11;12:742978. doi: 10.3389/fphar.2021.742978 (PMC8544810; doi:10.3389/fphar.2021.742978)

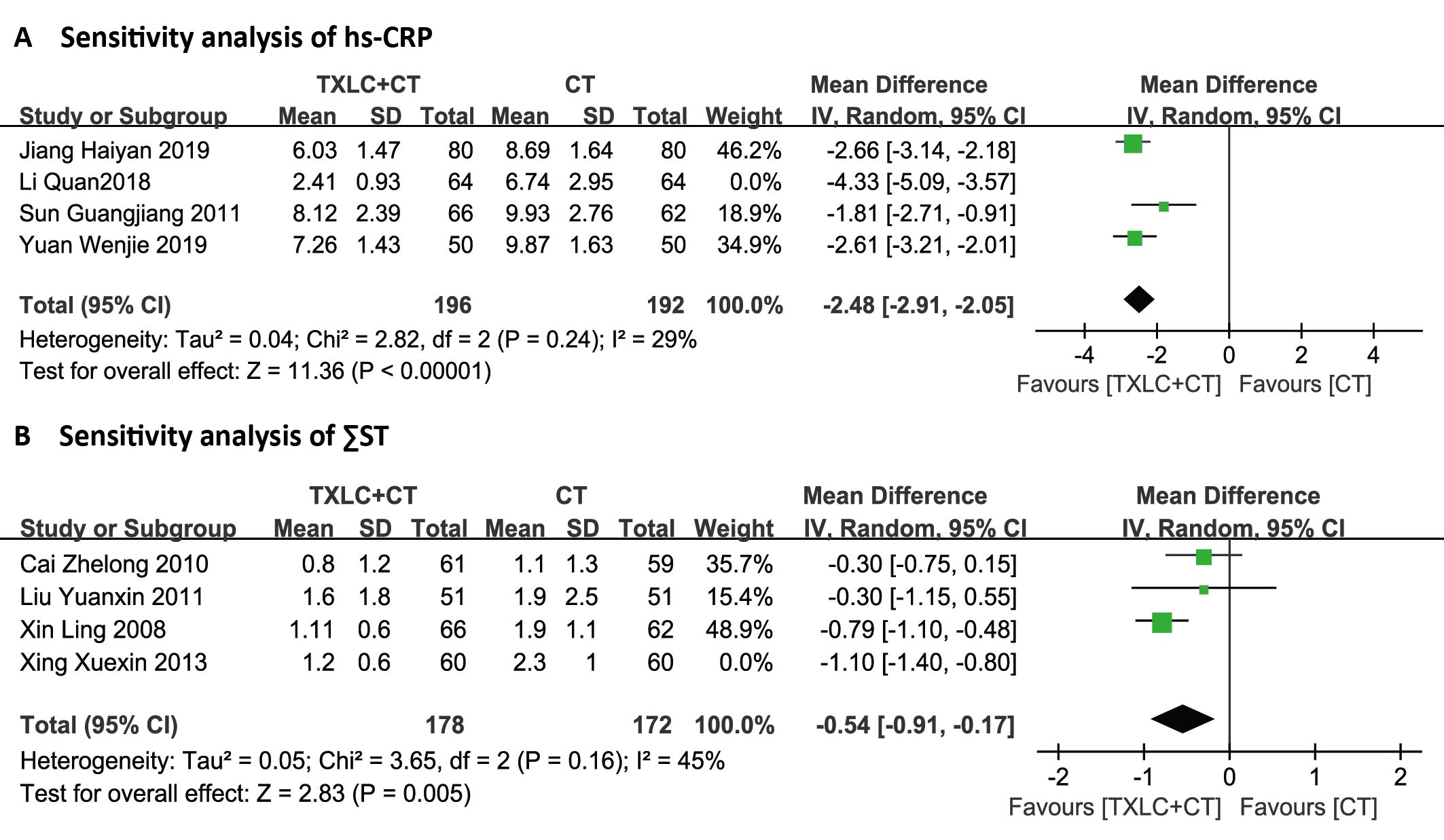

Supplement: Supplementary file 1 [file DataSheet1.zip › Supplementary material 4. Sensitivity analysis of hs-CRP and ∑ST using TXLC Plus CT vs. CT.jpg]
